# Executioner caspases degrade essential mediators of pathogen-host interactions to inhibit growth of intracellular *Listeria monocytogenes*

**DOI:** 10.1038/s41419-025-07365-x

**Published:** 2025-01-30

**Authors:** Marilyne Lavergne, Raffael Schaerer, Sara De Grandis, Safaa Bouheraoua, Oluwadamilola Adenuga, Tanja Muralt, Tiffany Schaerer, Léa Chèvre, Alessandro Failla, Patricia Matthey, Michael Stumpe, Dieter Kressler, Pierre-Yves Mantel, Michael Walch

**Affiliations:** 1 Faculty of Science and Medicine, Department of Oncology, Microbiology and Immunology, Anatomy unit, University of Fribourg, CH-1700 Fribourg, Switzerland; 2 Faculty of Science and Medicine, Department of Biology, Metabolomics and Proteomics Platform, University of Fribourg, CH-1700 Fribourg, Switzerland; 3 Christine Kühne – Center for Allergy Research and Education (CK-CARE), CH-7265 Davos Wolfgang, Switzerland

**Keywords:** Immune cell death, Infection

## Abstract

Cell death mediated by executioner caspases is essential during organ development and for organismal homeostasis. The mechanistic role of activated executioner caspases in antibacterial defense during infections with intracellular bacteria, such as *Listeria monocytogenes*, remains elusive. Cell death upon intracellular bacterial infections is considered altruistic to deprive the pathogens of their protective niche. To establish infections in a human host, *Listeria monocytogenes* deploy virulence mediators, including membranolytic listeriolysin O (LLO) and the invasion associated protein p60 (Iap), allowing phagosomal escape, intracellular replication and cell-to-cell spread. Here, by means of chemical and genetical modifications, we show that the executioner caspases-3 and -7 efficiently inhibit growth of intracellular *Listeria monocytogenes* in host cells. Comprehensive proteomics revealed multiple caspase-3 substrates in the *Listeria* secretome, including LLO, Iap and various other proteins crucially involved in pathogen-host interactions. *Listeria* secreting caspase-uncleavable LLO or Iap gained significant growth advantage in epithelial cells. With that, we uncovered an underappreciated defense barrier and a non-canonical role of executioner caspases to degrade virulence mediators, thus impairing intracellular *Listeria* growth.

## Introduction

To survive in the host, pathogenic bacteria evolved multiple effectors that allow specific interactions with the host to form a protective niche. The virulence strategy of *Listeria monocytogenes* (*Lm*) is characterized by uptake even in non-phagocytic cells, such as epithelial cells and fibroblasts, a process that is mediated by a group of surface proteins, called internalins (Inl) [1]. After uptake, they avoid lethal lysosomal degradation by a phagosomal escape mechanism, which is promoted by their key virulence mediator, listeriolysin O (LLO) in cooperation with two distinct phospholipases C [2]. In the cytosol, *Lm* specifically interact with the actin cytoskeleton, mediated by the actin assembly-inducing protein (ActA) [3] and the invasion associated protein p60 (Iap) [4], to gain motility for cell-to-cell spread, a process that is again mediated by LLO [5]. By successfully doing so, *Lm* cause a life-threatening disease in humans, particularly in the immunocompromised and during pregnancy [6].

Immune proteases have been recognized to display crucial internal barrier function in antibacterial defense. Neutrophil effector proteases have been demonstrated to be critical for the elimination of gram+ and gram- bacteria in in vitro and in vivo models [7–10]. These proteases were demonstrated to target bacterial proteins related to virulence [11], including LLO [12]. Our work revealed that the lymphocytic effector proteases, the granzymes, target bacterial proteins to inhibit their growth [13], including virulence mediators [14].

The induction of programed host cell death, particularly its lytic forms, necroptosis and pyroptosis, is widely recognized to act as an innate antibacterial defense barrier [15]. Less defined in antibacterial defense is the role of apoptosis [16], which is the immunologically silent, non-lytic form of programmed cell death, characterized by DNA fragmentation, chromatin condensation, membrane blebbing and cytoskeletal breakdown. This process relies on an intracellular cascade of the caspases. Initiator caspases-8 and -9 are activated by extrinsic factors, such as death receptor activation by tumor necrosis factor alpha (TNF-α) [17], intrinsic mediators, such as mitochondrial cytochrome C release [18], or by the granzymes [19]. Upon initiation, the executioner caspases-3, -6 and -7 are activated and they then cleave vital substrates resulting in apoptosis [20]. Apoptosis in the context of bacterial infections is considered as altruistic death, depriving intracellular bacteria of their protected niche. In this study, we have asked specifically if activated executioner caspases engage in more direct interactions with intracellular bacteria to inhibit their growth.

## Results

### Executioner caspases activity is induced by *Lm* infection and inhibits their intracellular growth

**Fig. 1:**
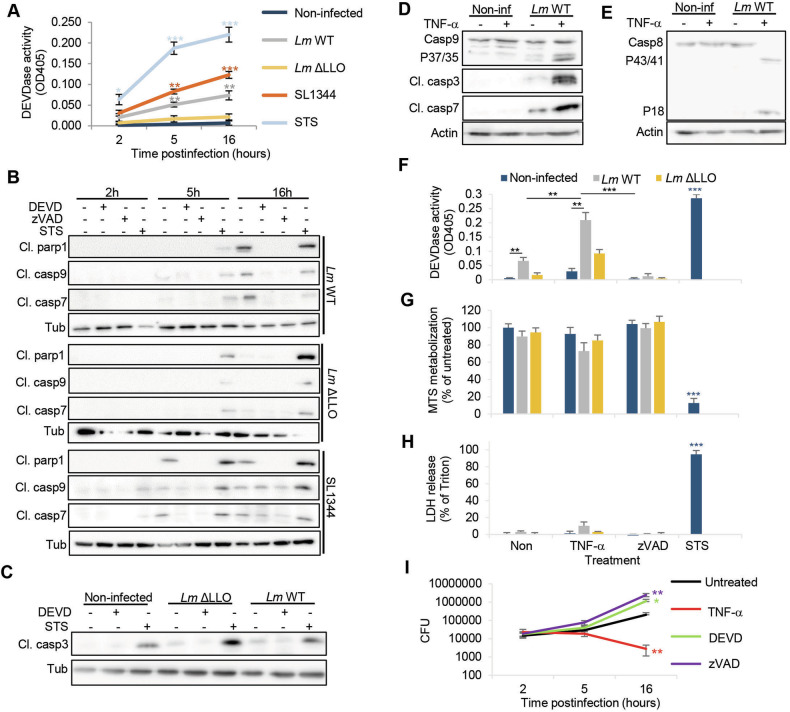
Executioner caspases activity is induced by *Listeria monocytogenes* infection in viable host cells that inhibits intracellular growth. **A** HeLa cells were treated with staurosporine (STS, 0.1 μg/ml) or infected with virulent *Lm* (WT, strain 10403S), listeriolysin O deficient *Lm* (ΔLLO) or *Salmonella enterica* serovar Typhimurium (SL1344) at a MOI of 1 for 1 h before extracellular bacteria were removed by gentamicin treatment. After further culture, the cells were lysed at indicated time points before caspase activity in the lysates was assessed using the chromogenic dye DEVD-pNA. Averages +/− SEM of three independent experiments are shown. HeLa cells infected with indicated bacteria as above +/− DEVD (20 μM), zVAD (20 μM) or STS (0.1 μg/ml) treatment were lysed at the indicated time points and their lysates were assessed by western blot for cleaved caspases-9 and -7 (Cl. casp9 and casp7), as well as the caspase-3-cleavage fragment of Parp1 (cl. parp1, **B**) or cleaved caspase-3 (cl. casp3) after 16 h (**C**). α−tubulin served as loading control. HeLa cells were infected with *Lm* WT as above or left non-infected (non-inf) for 16 h +/− TNF-α (10 ng/ml) before measuring caspase-9, caspase-3 and caspase-7 cleavage (**D**), as well as activation of caspase 8 (**E**) by immunoblot. In all panels, representative blots of three independent experiments are shown. DEVDase activity (**F**), MTS metabolic activity (**G**) and LDH release (**H**) were assessed in HeLa cells infected as above for 16 h with *Lm* WT, *Lm* ΔLLO or in uninfected cells +/− TNF-α (10 ng/ml) +/− zVAD (20 μM) treatment. Average +/− SEM of three independent experiments is shown. **I** HeLa cells were infected with *Lm* WT as above +/− DEVD-fmk (20 μM), zVAD (20 μM) or TNF-α (10 ng/ml) treatment. At indicated times, cells were lysed, and *Lm* were enumerated by CFU assay. Average +/− SEM of three independent experiments is presented. Asterisks indicate significant differences to untreated controls. *P*-values are * < 0.05, ** < 0.01 and *** < 0.005.

To experimentally confirm executioner caspases activity upon infection, we infected HeLa cells with WT or LLO-deficient *Lm* (both strain 10403S) and monitored executioner caspases (DEVDase) activity using the chromogenic caspase-3 and -7 substrate, Ac-DEVD-pNA (Fig. 1A). We also infected HeLa cells with *Salmonella enterica* serovar Typhimurium, in which caspase-3 activation was already mechanistically explored [21]. WT *Listeria* and *Salmonella* but not LLO-deficient *Lm* led to significant DEVDase activity 5 h postinfection that further increased after 16 hours. The colorimetric data were confirmed by western blot analysis using antibodies against the cleaved forms of the initiator caspase-9 (p35), executioner caspase-7 (p18), and of Parp1 (p89), which results upon caspase-3 cleavage [22] (Fig. 1B), as well as of the cleaved form of caspase-3 (Fig. 1C). Infections with virulent *Salmonella* and *Listeria* increased the signal intensity of cleaved Parp1 (rather weak for *Lm*), activated caspase-9 and caspase-7 already after 5 h and displayed full activation (compared to staurosporine, STS) after 16 hours. The LLO-deficient *Listeria* strain did not induce caspase-9 activity or Parp1 cleavage after 16 h, suggesting that it needs LLO for efficient caspase activation. Caspase activation was strongly reduced using the caspases-3 and -7 inhibitor DEVD-fmk or with pan-caspase inhibitor zVAD treatment indicating specificity of the detection. Caspase-3 activation (p17 fragment) upon virulent *Lm* infection alone was less efficient, only resulting in weak signal of cleaved caspase-3 after 16 h (Fig. 1C). As the death receptor ligand TNF-α is known to induce caspase-mediated apoptosis in intrinsically stressed HeLa cells, e.g. by protein synthesis inhibition [23, 24], and mice lacking TNF-α quickly succumb to *Lm* infections [25], we infected HeLa cells with *Lm* in presence of TNF-α for 16 h and measured caspase activation by immunoblot. Indeed, treatment with TNF-α markedly increased the activation of caspase-9, -3 and -7 (Fig. 1D). As a possible explanation for this phenomenon, we found full caspase-8 activation only in conditions of *Lm* infection with TNF-α indicating intrinsic and extrinsic apoptotic signals simultaneously present in these cells that drive downstream caspase activation (Fig. 1E).

Interestingly, despite significant DEVDase activity in *Lm*-infected HeLa cells that was further enhanced by TNF-α and abolished by zVAD (Fig. 1F), we found no significant decrease of MTS metabolization (Fig. 1G) or release of LDH from infected cells after 16 h (Fig. 1H). This contrasted with STS treatment, which lysed the cells completely after 16 h.

Most importantly, the inhibition of executioner caspase activity in *Lm*-infected HeLa cells with caspase-3/7 specific DEVD-fmk or pan-caspase inhibitor zVAD increased growth of intracellular *Lm* whereas the treatment with TNF-α reduced the bacterial burden as compared to untreated cells (Fig. 1I).

### *Lm* infection in presence of TNF-α results in high cytoplasmic DEVDase activity without inducing host cell death

**Fig. 2:**
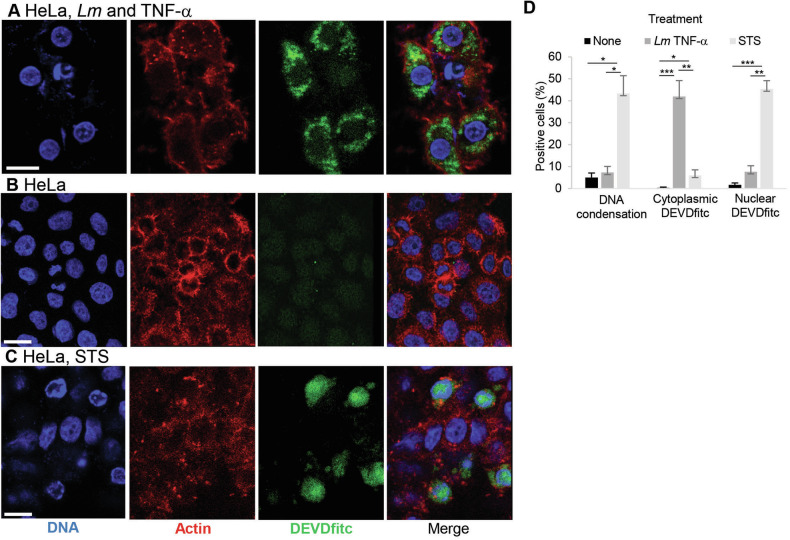
Cytoplasmic DEVDase activity is induced by *Lm* infection in presence of TNF-α. HeLa cells were infected with *Lm* at a MOI of 1 in presence of TNF-a (**A**), left without any manipulations (**B**), or treated with STS (0.1 mg/ml) (**C**) for 7 h before staining the cells with FITC-DEVD-fmk for another hour. After fixation, the cells were counterstained with Hoechst and 647-phallodin, then analyzed by confocal microscopy. In all panels, bars are 20 μm. (**D**), DNA condensation, cytoplasmic and nuclear DEVDfitc was quantified by counting five visible fields (n ~ 50) in three independent experiments. Presented are average +/− SEM. Significant differences are indicated. *P*-values are * < 0.05, ** < 0.01 and *** < 0.005.

To assess morphological signs of cell death upon *Lm* infection in presence of the proapoptotic agent TNF-α, we used fluorescent DEVD to indicate active caspases by microscopy. Strikingly, around half of the cells displayed bright cytoplasmic DEVD staining after 7 h upon *Lm* infection and TNF-α treatment (Fig. 2A, D). However, the nuclear morphology and cytoskeleton organization of the infected cells did not display obvious apoptotic features, similar to negative control HeLa cells (Fig. 2B). This was in stark contrast to STS treatment, leading to nuclear caspase translocation [26], as well as chromatin condensation and actin cytoskeleton breakdown [27] (Fig. 2C, D).

**Fig. 3:**
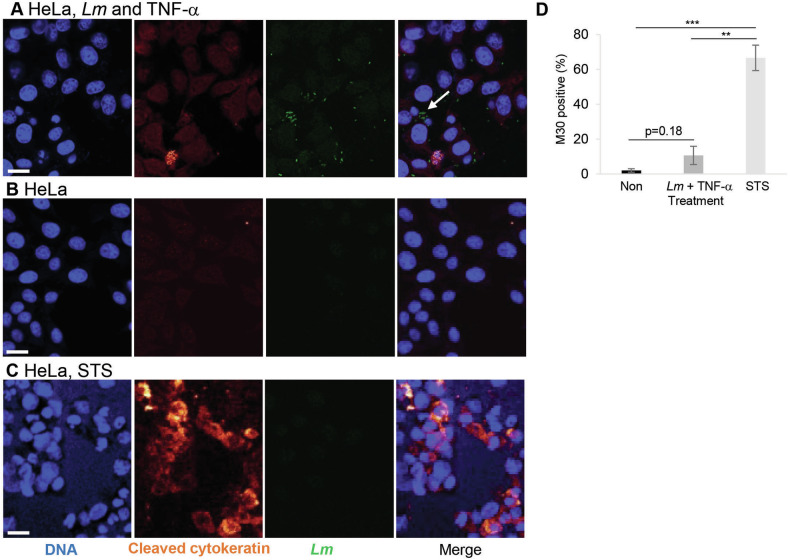
*Lm* infection in presence of TNF-α does not lead to caspase-mediated cytokeratin-18 cleavage. HeLa cells were infected with green fluorescent *Lm* (white arrow) in presence of TNF-α (**A**), left untreated (**B**), or treated with STS (0.1 mg/ml) (**C**) for 7 h. After methanol fixation, cytokeratin-18 cleavage was assessed using the M30 Cytodeath® antibody and Hoechst counter staining by microscopic analysis. (**D**), M30 positive cells were quantified by counting five visible fields (*n* ~ 50) in three independent experiments. Depicted are representative images from three independent experiments. Presented are average +/− SEM. Significant differences are indicated. *P*-values are * < 0.05, ** < 0.01 and *** < 0.005.

In addition, in *Lm*-infected cells treated with TNF-α, only a few cells bound the Cytodeath® M30 antibody detecting cleaved cytokeratin-18 in early apoptotic cells [28] (Fig. 3A, D) as compared to untreated control cells (Fig. 3B). Even in cells showing active *Lm* proliferation, cytokeratin-18 remained unaffected (arrow in Fig. 3A). This was again in sharp contrast to STS treatment where most of the cells stained positive for M30 (Fig. 3C, D).

### Host cells lacking executioner caspases are less resistant to bacterial infection

**Fig. 4:**
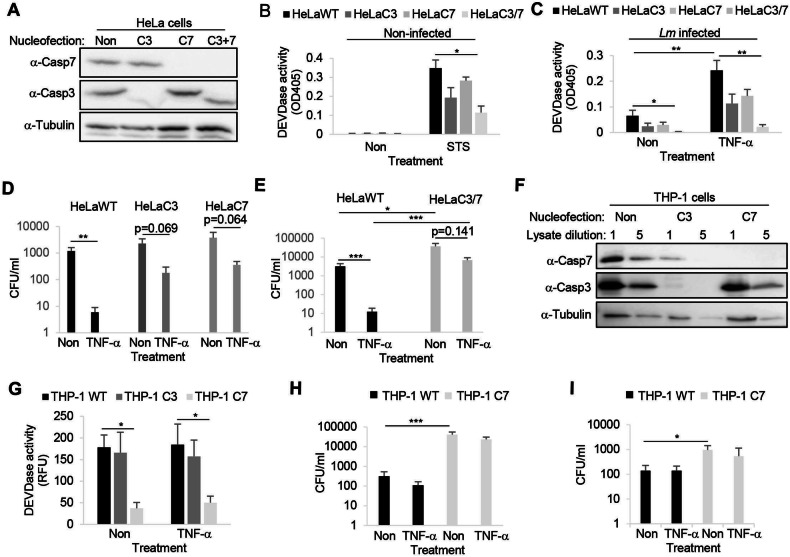
Host cells depleted of executioner caspases are less resistant to intracellular bacterial growth. Parental HeLa or commercial caspase-3 KO (C3) cells (Abcam) were nucleofected with Cas9/guide RNA ribonucleoproteins (RNPs) targeting caspase-7 before enriching for deletion lines by limited dilutions. Knockout was confirmed by immunoblot using anti caspase-3, caspase-7 and α-tubulin antibodies (**A**). **B** caspase-deficient or parental HeLa lines were treated with staurosporine (STS) for 7 h before lysis and measuring DEVDase activity using DEVD-pNA. Indicated HeLa lines were infected with WT *Listeria* at MOI 0.1 for 16 h +/− TNF-α before the cells were lysed and DEVDase activity was measured (**C**), and CFU were calculated from bacterial growth curves (**D** and **E**), as shown in Figure S1. Averages +/− SEM of three independent experiments are shown. THP-1 cells were nucleofected with Cas9/guide RNPs targeting caspase-3 or -7 before cloning by limited dilutions and assessment by western blot (**F**). Cells of indicated genotype were infected with *Lm* at the MOI of 0.1 for 16 h +/− TNF-α, then the cells were lysed and DEVDase activity was assessed using fluorescent DEVD-fmk (**G**), and CFU were determined as above (**H**). WT and caspase-7 KO THP-1 cells were additionally infected with SL1344 at a MOI of 0.001 + /- TNF-α for 16 h before intracellular CFU were calculated from growth curves as shown in Figure S1G (**I**). Averages +/− SEM of three independent experiments are shown and significant differences between groups are indicated by asterisks. P-values are * < 0.05, ** < 0.01 and *** < 0.005.

As the chemical inhibition of caspases is prone to off-target effects, executioner caspases were deleted in HeLa cells using CRISPR/Cas9 technology. For this purpose, HeLa cells (a commercial caspase-3 knockout (KO) and the corresponding parental line) were nucleofected with Cas9/guide RNA ribonucleoproteins (RNPs) directed to caspase-7 before cloning deletion lines by limited dilutions (Fig. 4A). First, the engineered lines were assessed for their levels of DEVDase activity in response to STS (Fig. 4B). While there was a clear (yet not significant) reduction in DEVDase activity in the single KO lines, DEVDase activity decreased in the *CASP3*−/− and *CASP7*−/− HeLa line as compared to the parental cells. This pattern was also displayed upon infection with *Lm*, where again caspase activity was only statistically significantly reduced in *CASP3*−/− and *CASP7*−/− cells; this was particularly obvious in host cells that were simultaneously treated with TNF-α (Fig. 4C). To evaluate the change DEVDase activity in *CASP3*−/− or *CASP7*−/− cells, we assessed intracellular *Lm* growth with and without TNF-α treatment. In these experiments, CFUs were calculated from bacterial growth curves as illustrated in the supplementary Figures S1. While TNF-α significantly reduced the bacterial burden in the parental HeLa line (WT), bacterial growth was not significantly affected by TNF-α in caspase deleted host cells (Fig. 4D). Only *CASP3*−/− and *CASP7*−/− HeLa cells were significantly less resistant to intracellular *Lm* growth than the parental line in absence of TNF-α (Fig. 4E). Of note, though executioner activity in HeLa cells was highly increased upon gram- *Salmonella* infections (Fig. 1A), their growth in *CASP3*−/− and *CASP7*−/− cells was not significantly affected (data not shown), suggesting strain specificity of this caspase-mediated defense mechanism in epithelial cells. To study the impact of the host cell type, we additionally aimed to delete caspase-3 and -7 in the monocyte-like human cell line THP-1. While the depletion of caspase-7 was complete, a weak band in the caspase-3 immunoblot still appeared after multiple rounds of nucleofection and limited dilution cloning (Fig. 4F) that was also reflected by the largely unchanged DEVDase activity upon *Lm* infection in this line (Fig. 4G). In contrast to HeLa cells, simultaneous TNF-α treatment did not alter DEVDase activity neither in the WT cells nor the KO lines. As the depletion of caspase-3 in THP-1 cells was obviously incomplete, we only tested the *CASP7−/−* THP-1 cells for intracellular bacteria growth. Indeed, we found a significantly increased bacterial burden in, *Lm-* (Fig. 4H) and surprisingly also *Salmonella*-infected *CASP7−/−* THP-1 cells (Fig. 4I), that was again not affected by simultaneous TNF-α treatment.

### Caspase-3 degrades *Listeria* supernatant proteins involved in pathogen-host interactions

**Fig. 5:**
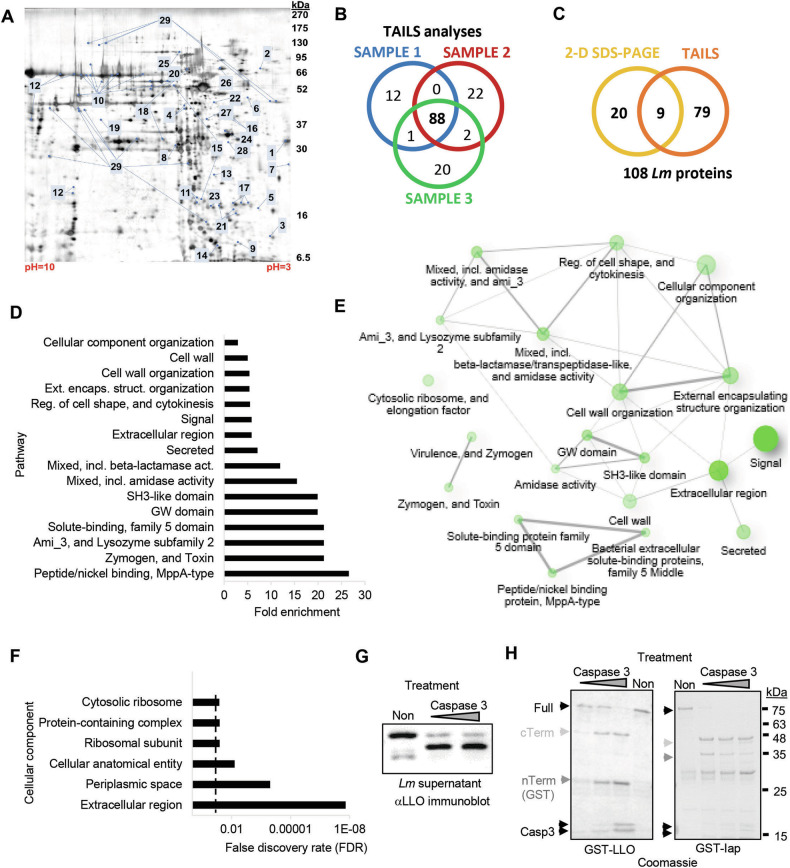
Caspase-3 targets multiple vital *Lm* pathways related to pathogen-host interactions. Cell-free *Lm* supernatants were treated with purified caspase-3 before analysis by comparative 2D SDS-PAGE (**A**), analyzed spots are indicated on the non-treated gel or by TAILS degradomics (**B**), Venn Diagram. The overlap in proteins is depicted in (**C**). All proteins are listed in Table 1 and the protein network analysis is indicated in Figure S2. Gene ontology analysis using ShinyGO 0.80 software revealed several significantly enriched pathways (**D**) that demonstrated to be highly interconnected (**E**). Two pathways (green nodes) are considered connected if they share at least 20% genes. Darker nodes represent more significantly enriched gene sets and bigger nodes are larger gene sets. Thicker edges indicate more overlapped genes. (**F**) Gene ontology analysis indicates significant enrichment in cellular component. Dashed line shows the FDR cut-off p-value of 0.05. Caspase-3-mediated degradation (caspase-3/*Lm* protein weight ratios from 1/1 to 1/100 for 4 h) of LLO in *Lm* supernatant was confirmed by western blot (**G**), and of purified GST-tagged LLO and Iap by Coomassie stained SDS-PAGE (**H**). Full-length protein, cleavage fragments and caspase-3 are indicated by arrow heads.

We next studied if the proteolytic activity of executioner caspases – and with that, bacterial substrate degradation – might contribute to bacterial growth inhibition. Caspase accessibility was hypothesized to favor degradation of bacterial proteins that are released into the cytoplasm. Therefore, we performed unbiased proteomics approaches to identify caspase-3 substrates in cell-free *Lm* supernatants. Comparative 2-dimensional (2D) SDS-PAGE identified 29 proteins whose intensities changed by at least a factor 2 upon caspase-3 treatment in three replicate analyses (Fig. 5A). To backup these findings using an alternative approach, we additionally analyzed the caspase-3 degradome in *Lm* supernatants by terminal amine isotopic labeling of substrates (TAILS) [29]. TAILS analysis revealed 88 supernatant proteins that were detected as cleaved in three independent replicate samples (Fig. 5B). To our surprise, the overlap of the two analyses was only partial (9 proteins, Fig. 5C), presumably due to major differences in the sensitivities of the assays. All substrates of the two approaches and the potential cleavage sites according to the TAILS analysis are listed in Table 1. The 9 overlapping proteins are at the top of the list, including the well-established *Lm* virulence factors listeriolysin O (LLO) [5] and the broad range phospholipase C (PlcB) [30], essential for cell-to-cell spread. As the substrate selection criteria in the two approaches were highly stringent (found in 3 replicate samples), we run the bioinformatics analyses with the pooled substrate list of 108 proteins. We first screened the substrate list for pathways that are significantly enriched according to their false discovery rate (FDR) and ordered them by fold enrichment (Fig. 5D). The top enriched pathways include ATP-binding cassette (ABC) transporter complexes, PrfA-dependent virulence factors and peptidoglycan hydrolases, all essential for full virulence in vivo [31–33]. A pathway network analysis revealed a high interconnectivity between the enriched pathways (Fig. 5E), clearly indicating targeted substrate selection by caspase-3. Gene Oncology (GO) analysis of the cellular components showed, as expected, the extracellular region to be at the top of the list (Fig. 5F). However, the enrichment of membrane proteins and cytoplasmic proteins in a proteomics analysis of cell-free bacterial supernatant was unexpected. This was also indicated in the protein-protein interaction (PPI) network analysis by the STRING software with most proteins being neither extracellular nor periplasmic (Figure S2) [34]. The PPI analysis though with an enrichment p-value of 1.08e-12 proofed to be highly significant, contracting a random set of proteins.

**Table 1:** Caspase-3 substrates in *Lm* supernatants assessed by TAILS and 2D SDS-PAGE proteomics.

*Lm* 10403S protein Name	*Lm* 10403S protein Accession number	Name of the homologous gene in *Lm* serovar 1/2a (strain ATCC BAA-679 / EGD-e)	Position of the cleavage (in *Lm* 10403S protein)	Cleavage sequence (in *Lm* 10403S protein)
Thiol-activated cytolysin (listeriolysin O, LLO)	A0A0H3GD84	lmo0202	A0A0H3GD84 [63-89]	[D].KYIQGLDYNKNNVLVYHGDAVTNVPPR.[K]
Phosphatidylcholine cholinephosphohydrolase (PlcB)	A0A0H3G8P1	lmo0205	A0A0H3G8P1 [205-220]	[D].TIKHNYQATEDMVAKR.[F]
	A0A0H3G8P1		A0A0H3G8P1 [229-236]	[D].WLYENAKR.[A]
Fumarate reductase flavoprotein subunit	A0A0H3GDC3	lmo0355	A0A0H3GDC3 [160-178]	[D].SMDIKLNNLTITGGMSEKR.[T]
	A0A0H3GDC3		A0A0H3GDC3 [458-479]	[D].GKPITGLFAAGEVTGGLHGENR.[I]
	A0A0H3GDC3		A0A0H3GDC3 [160-178]	[D].SMDIKLNNLTITGGMSEKR.[T]
	A0A0H3GDC3		A0A0H3GDC3 [172-178]	[T].GGMSEKR.[T]
Peptidase_M23 domain-containing protein	A0A0H3GFN8	lmo2504	A0A0H3GFN8 [144-151]	[D].DFKELVDR.[V]
Peptidoglycan bound protein	A0A0H3GGA1	lmo2714	A0A0H3GGA1 [107-116]	[D].YTDTKLTNYR.[L]
Peptide/nickel transport system substrate-binding protein	A0A0H3GJB6	lmo2196	A0A0H3GJB6 [485-503]	[D].YDKILNDASVTYAADDQKR.[W]
Transcriptional regulator LytR	A0A0H3GKG2	lmo2518	A0A0H3GKG2 [198-224]	[D].SKDNAGAITLKKGPQHLNAEQALALAR.[T]
60 kDa chaperonin	A0A0H3GLZ0	lmo2068	A0A0H3GLZ0 [190-195]	[E].GMQFDR.[G]
D-glutamyl-L-m-Dpm peptidase P45	A0A0H3GN80	lmo2505	A0A0H3GN80 [222-230]	[D].KAAKEATAR.[A]
Quinol oxidase subunit 2	A0A0H3G8T0	lmo0013	A0A0H3G8T0 [198-205]	[D].EVGTYKGR.[N]
NusG_II domain-containing protein	A0A0H3G991	lmo0408	A0A0H3G991 [69-85]	[T].IKGKGAQYNLMEVDGER.[I]
Cysteine synthase	A0A0H3G9A7	lmo0223	A0A0H3G9A7 [234-253]	[D].TKVYDGILKVSSEDALETAR.[E]
Uncharacterized protein	A0A0H3G9Q4	lmo0592	A0A0H3G9Q4 [99-111]	[D].AGEVKDALNVFER.[T]
Glycerophosphoryl diester phosphodiesterase	A0A0H3G9S8	lmo0616	A0A0H3G9S8 [331-342]	[T].LYEPNTKIIAHR.[G]
Lipoprotein	A0A0H3G9T8	lmo0366	A0A0H3G9T8 [225-233]	[D].WTGFHVIER.[A]
Internalin B	A0A0H3G9Y9	lmo0434	A0A0H3G9Y9 [331-343]	[D].GTVIKTKVEAGTR.[I]
Tagatose 1,6-diphosphate aldolase	A0A0H3GA75	lmo0539	A0A0H3GA75 [284-297]	[D].GIEVYGKQGDDALR.[E]
Spermidine/putrescine transport system permease	A0A0H3GAF1	lmo0808	A0A0H3GAF1 [168-173]	[T].LIQASR.[D]
Sulfatase domain-containing protein	A0A0H3GAG2	lmo0644	A0A0H3GAG2 [576-588]	[D].GTNKELSSDVKKR.[F]
Uncharacterized protein	A0A0H3GBD8	lmo0944	A0A0H3GBD8 [19-26]	[D].VANGNGIR.[L]
GW domain-containing protein	A0A0H3GBE7	lmo1215	A0A0H3GBE7 [97-107]	[D].GKWIEINANFR.[K]
Uncharacterized protein	A0A0H3GBP9	lmo1076	A0A0H3GBP9 [35-46]	[D].SSEQEDNTEVAR.[E]
ABC transport system	A0A0H3GBZ4	lmo1388	A0A0H3GBZ4 [80-101]	[D].GYNYLQSASEADYKTNLNTAVR.[S]
	A0A0H3GBZ4		A0A0H3GBZ4 [92-101]	[D].YKTNLNTAVR.[S]
	A0A0H3GBZ4		A0A0H3GBZ4 [277-290]	[D].GKDYNVTLTSEIKR.[V]
Signal peptidase I	A0A0H3GC68	lmo1271	A0A0H3GC68 [130-145]	[D].GGKIPKDTYFVLGDNR.[R]
	A0A0H3GC68		A0A0H3GC68 [130-146]	[D].GGKIPKDTYFVLGDNRR.[A]
DNA gyrase subunit B	A0A0H3GCB7	lmo0006	A0A0H3GCB7 [439-448]	[D].SAGGSAKQGR.[D]
Universal stress protein	A0A0H3GCF8	lmo1580	A0A0H3GCF8 [14-20]	[D].GSKEAER.[A]
Protease IV	A0A0H3GCG3	lmo1585	A0A0H3GCG3 [78-100]	[D].SGSLFSEAGYNHSFFMQQLEQVR.[N]
Uncharacterized protein	A0A0H3GCN8	lmo0130	A0A0H3GCN8 [137-144]	[D].EGLPEYKR.[I]
	A0A0H3GCN8		A0A0H3GCN8 [420-429]	[D].FAMTNNGGIR.[S]
	A0A0H3GCN8		A0A0H3GCN8 [75-94]	[D].NATNSFLQANPGATTDNAIR.[V]
	A0A0H3GCN8		A0A0H3GCN8 [290-308]	[D].LVLAGHNHQYTNGLVGKTR.[I]
Peptide/nickel transport system substrate-binding protein	A0A0H3GCP2	lmo0135	A0A0H3GCP2 [224-234]	[D].GKPKLDKVTFR.[I]
	A0A0H3GCP2		A0A0H3GCP2 [110-135]	[D].GKPLTADDVVFTVNSILDTKQNSPNR.[G]
	A0A0H3GCP2		A0A0H3GCP2 [207-217]	[E].YKTGEYVSLER.[F]
G5 domain-containing protein	A0A0H3GCT9	lmo0186	A0A0H3GCT9 [77-98]	[D].EIAPGKNAEIKDGMEIKYLPAR.[Q]
	A0A0H3GCT9		A0A0H3GCT9 [77-98]	[D].EIAPGKNAEIKDGMEIKYLPAR.[Q]
	A0A0H3GCT9		A0A0H3GCT9 [141-153]	[D].TKLKNGLEVNINR.[A]
	A0A0H3GCT9		A0A0H3GCT9 [89-98]	[D].GMEIKYLPAR.[Q]
Putative septation protein SpoVG	A0A0H3GCV1	lmo0196	A0A0H3GCV1 [27-35]	[D].GEFVIHDIR.[V]
Aspartyl/glutamyl-tRNA(Asn/Gln) amidotransferase subunit B	A0A0H3GCW2	lmo1754	A0A0H3GCW2 [279-288]	[D].LFIDDAWKER.[I]
50S ribosomal protein L25	A0A0H3GCW8	lmo0211	A0A0H3GCW8 [45-53]	[D].SLELIKAVR.[D]
YycH protein	A0A0H3GD67	lmo0289	A0A0H3GD67 [252-274]	[D].GSSVIEMDTDNKVLEYVNPSQER.[T]
DivIC protein	A0A0H3GD99	lmo0217	A0A0H3GD99 [85-104]	[D].SLNEQIKKLHNDDYIAKLAR.[S]
	A0A0H3GD99		A0A0H3GD99 [85-104]	[D].SLNEQIKKLHNDDYIAKLAR.[S]
Probable transcriptional regulatory protein LMRG_00061	A0A0H3GDD6	lmo0369	A0A0H3GDD6 [210-217]	[G].EDLEKFER.[L]
Peptidoglycan-N-acetylglucosamine deacetylase PgdA	A0A0H3GDH9	lmo0415	A0A0H3GDH9 [125-135]	[K].AVQSEYVKEGR.[T]
Probable succinyl-diaminopimelate desuccinylase	A0A0H3GDI0	lmo0265	A0A0H3GDI0 [158-168]	[D].GLIIGEPSGHR.[I]
LytR_cpsA_psr domain-containing protein	A0A0H3GDK2	lmo0443	A0A0H3GDK2 [191-199]	[D].GKTLLQYAR.[F]
10 kDa chaperonin	A0A0H3GDS3	lmo2069	A0A0H3GDS3 [29-46]	[D].SAKEKPQSGKIVAVGSGR.[V]
Manganese-binding lipoprotein mntA	A0A0H3GDS9	lmo1847	A0A0H3GDS9 [282-298]	[D].STAKKGEVGDTYLEMMR.[Y]
4-hydroxy-tetrahydrodipicolinate reductase	A0A0H3GDZ1	lmo1907	A0A0H3GDZ1 [159-175]	[D].APSGTGVKTAEMMAETR.[E]
Histidine kinase	A0A0H3GE39	lmo1947	A0A0H3GE39 [36-41]	[E].KNNITR.[I]
Flagellar hook protein FlgE	A0A0H3GEC2	lmo0697	A0A0H3GEC2 [79-89]	[D].YTAGSPTSTGR.[N]
Phage capsid protein	A0A0H3GEG6	lmo2296	A0A0H3GEG6 [262-272]	[D].ASKGNDQVFTR.[R]
Aminotransferase	A0A0H3GEK9	lmo2370	A0A0H3GEK9 [20-27]	[D].GAEELFGR.[K]
Peptidoglycan bound protein	A0A0H3GES0	lmo0842	A0A0H3GES0 [364-383]	[D].SYNYTKSYSVVNGSNSLDSR.[G]
CBM6 domain-containing protein	A0A0H3GET7	lmo2446	A0A0H3GET7 [1013-1022]	[D].GASDYTMEVR.[Y]
30S ribosomal protein S5	A0A0H3GFB3	lmo2615	A0A0H3GFB3 [7-15]	[D].GNKLDLEER.[V]
Phosphoglycerol transferase	A0A0H3GFC6	lmo0927	A0A0H3GFC6 [446-452]	[D].TYFQTAR.[Y]
	A0A0H3GFC6		A0A0H3GFC6 [617-625]	[D].SVLQGDLLR.[F]
	A0A0H3GFC6		A0A0H3GFC6 [442-452]	[D].SSVDTYFQTAR.[Y]
FeS assembly protein SufD	A0A0H3GFE5	lmo2414	A0A0H3GFE5 [370-380]	[D].VVAGHAASVGR.[V]
Uncharacterized protein	A0A0H3GFH4	lmo2444	A0A0H3GFH4 [179-187]	[D].GGIFDGGVR.[F]
Potassium-transporting ATPase KdpC subunit	A0A0H3GFI3	lmo2680	A0A0H3GFI3 [82-101]	[D].GVATNLNPTSEEQKQLVEKR.[I]
Glyceraldehyde-3-phosphate dehydrogenase	A0A0H3GFI9	lmo2459	A0A0H3GFI9 [189-197]	[D].APHPKGDFR.[R]
HTH lacI-type domain-containing protein	A0A0H3GFM6	lmo1030	A0A0H3GFM6 [1-8]	[-].MKLEDIAR.[L]
SSD domain-containing protein	A0A0H3GFS0	lmo1226	A0A0H3GFS0 [135-141]	[D].ASVKSIR.[N]
	A0A0H3GFS0		A0A0H3GFS0 [127-141]	[A].SLTVDDTDASVKSIR.[N]
Peptide/nickel transport system substrate-binding protein	A0A0H3GFV1	lmo2569	A0A0H3GFV1 [98-107]	[D].GGKTLIFKIR.[E]
Signal peptidase I	A0A0H3GFV8	lmo1269	A0A0H3GFV8 [50-63]	[D].GEHLFINKVSDPKR.[F]
	A0A0H3GFV8		A0A0H3GFV8 [50-63]	[D].GEHLFINKVSDPKR.[F]
30S ribosomal protein S19	A0A0H3GG04	lmo2628	A0A0H3GG04 [68-78]	[V].GHKLGEFAPTR.[T]
Elongation factor Tu	A0A0H3GG29	lmo2653	A0A0H3GG29 [198-205]	[D].SYIPTPER.[D]
	A0A0H3GG29		A0A0H3GG29 [111-117]	[D].GPMPQTR.[E]
	A0A0H3GGA1		A0A0H3GGA1 [110-116]	[D].TKLTNYR.[L]
	A0A0H3GGA1		A0A0H3GGA1 [110-116]	[D].TKLTNYR.[L]
	A0A0H3GGA1		A0A0H3GGA1 [100-116]	[D].FTATLTDYTDTKLTNYR.[L]
Transcription elongation factor GreA	A0A0H3GGF0	lmo1496	A0A0H3GGF0 [53-64]	[D].SAKDEQAFVEGR.[I]
N-acetylmuramoyl-L-alanine amidase	A0A0H3GGH0	lmo1521	A0A0H3GGH0 [179-184]	[D].DSTNIR.[N]
	A0A0H3GGH0		A0A0H3GGH0 [235-242]	[D].KGQTSSPR.[S]
	A0A0H3GGH0		A0A0H3GGH0 [351-370]	[D].KSLAESINTTLGKDLPTTNR.[G]
Transketolase	A0A0H3GGH6	lmo1305	A0A0H3GGH6 [7-14]	[D].SLAVNTIR.[T]
Glutamyl-tRNA(Gln) amidotransferase subunit A	A0A0H3GH17	lmo1755	A0A0H3GH17 [236-245]	[D].STSINQPVER.[F]
Multifunctional fusion protein	A0A0H3GH33	lmo1527	A0A0H3GH33 [65-72]	[D].TVTSLDKR.[V]
Cell shape-determining protein MreC	A0A0H3GH53	lmo1547	A0A0H3GH53 [65-82]	[D].GVVDLKNTYTENQHLKER.[L]
Actin-assembly inducing protein ActA	A0A0H3GH64	lmo0204	A0A0H3GH64 [412-424]	[D].DENSETTEEEIDR.[L]
	A0A0H3GH64		A0A0H3GH64 [36-60]	[D].SSLNTDEWEEEKTEEQPSEVNTGPR.[Y]
	A0A0H3GH64		A0A0H3GH64 [167-174]	[D].KPTKANKR.[K]
	A0A0H3GH64		A0A0H3GH64 [412-424]	[D].DENSETTEEEIDR.[L]
	A0A0H3GH64		A0A0H3GH64 [125-132]	[D].RPTLQVER.[R]
30S ribosomal protein S2	A0A0H3GHH8	lmo1658	A0A0H3GHH8 [15-21]	[V].HFGHQTR.[R]
Carboxyl-terminal processing protease	A0A0H3GI96	lmo1851	A0A0H3GI96 [171-187]	[D].GKSVKGDTATEATQKIR.[G]
	A0A0H3GI96		A0A0H3GI96 [440-452]	[D].GLYDTDTEYAVQR.[F]
Flagellin	A0A0H3GID7	lmo0690	A0A0H3GID7 [42-51]	[D].DAAGLAVVTR.[M]
UPF0302 protein LMRG_01068	A0A0H3GIF4	lmo1921	A0A0H3GIF4 [163-171]	[D].EAAFHKLVR.[I]
Uncharacterized protein	A0A0H3GJ46	lmo2522	A0A0H3GJ46 [211-216]	[D].LNDNSR.[V]
	A0A0H3GJB6		A0A0H3GJB6 [127-132]	[D].YVYSWR.[R]
	A0A0H3GJB6		A0A0H3GJB6 [263-277]	[D].SGTGLNLYNTDKVDR.[T]
Pyruvate dehydrogenase E1 component subunit alpha	A0A0H3GJB7	lmo1052	A0A0H3GJB7 [238-252]	[D].GMDPLAVYAVTKFAR.[E]
50S ribosomal protein L18	A0A0H3GJD7	lmo2616	A0A0H3GJD7 [79-90]	[D].AASKVGELVAKR.[A]
FAD:protein FMN transferase	A0A0H3GJF7	lmo2636	A0A0H3GJF7 [56-72]	[L].KIYDKGKEDVLDKGFDR.[I]
	A0A0H3GJF7		A0A0H3GJF7 [256-262]	[V].TSGIYER.[Y]
30S ribosomal protein S12	A0A0H3GJH5	lmo2656	A0A0H3GJH5 [85-96]	[G].HNLQEHSVVLIR.[G]
Polar amino acid transport system ATP-binding protein	A0A0H3GJI6	lmo2251	A0A0H3GJI6 [190-200]	[M].VVVTHEMGFAR.[E]
Dihydroxyacetone kinase L subunit	A0A0H3GJL3	lmo2696	A0A0H3GJL3 [102-119]	[D].AVGLTKVIEAGLEGIEKR.[G]
dITP/XTP pyrophosphatase	A0A0H3GJR4	lmo1239	A0A0H3GJR4 [80-88]	[D].GAPGVYSAR.[Y]
Multiple sugar transport system substrate-binding protein	A0A0H3GJY9	lmo2839	A0A0H3GJY9 [119-134]	[D].SVGEDKYYEGATNLVR.[S]
Uncharacterized protein	A0A0H3GK85	lmo1438	A0A0H3GK85 [62-71]	[D].NVTVSKNVPR.[G]
	A0A0H3GK85		A0A0H3GK85 [36-41]	[F].SVLILR.[L]
	A0A0H3GKG2		A0A0H3GKG2 [201-224]	[D].NAGAITLKKGPQHLNAEQALALAR.[T]
N-acetylmuramoyl-L-alanine amidase	A0A0H3GKJ7	lmo2558	A0A0H3GKJ7 [41-51]	[D].GQATYIPKGVR.[D]
	A0A0H3GKJ7		A0A0H3GKJ7 [40-51]	[V].DGQATYIPKGVR.[D]
	A0A0H3GKJ7		A0A0H3GKJ7 [31-51]	[A].ASIDPVQKVDGQATYIPKGVR.[D]
50S ribosomal protein L14	A0A0H3GKP3	lmo2622	A0A0H3GKP3 [10-17]	[K].VADNSGAR.[E]
Peptidoglycan bound protein	A0A0H3GKR6	lmo1666	A0A0H3GKR6 [36-45]	[D].DISPEISDNR.[F]
Polar amino acid transport system substrate-binding protein	A0A0H3GKX3	lmo1738	A0A0H3GKX3 [227-232]	[D].FAVGMR.[K]
	A0A0H3GKX3		A0A0H3GKX3 [182-191]	[D].TAFIDLNNKR.[I]
	A0A0H3GLZ0		A0A0H3GLZ0 [25-35]	[N].AVKVTLGPKGR.[N]
	A0A0H3GLZ0		A0A0H3GLZ0 [488-498]	[D].AGIVDPTKVTR.[S]
Polar amino acid transport system substrate-binding protein	A0A0H3GMT4	lmo2349	A0A0H3GMT4 [132-146]	[D].SNNSINSTKDLAGKR.[V]
	A0A0H3GMT4		A0A0H3GMT4 [132-146]	[D].SNNSINSTKDLAGKR.[V]
Enolase	A0A0H3GN27	lmo2455	A0A0H3GN27 [295-304]	[D].WDGFKLLTER.[I]
	A0A0H3GN80		A0A0H3GN80 [206-220]	[D].QASAENAKAGLVSER.[D]
	A0A0H3GN80		A0A0H3GN80 [206-220]	[D].QASAENAKAGLVSER.[D]
DD-transpeptidase	A0A0H3GDX6	lmo1892	A0A0H3GDX6 [163-170]	[D].YTNKTLAR.[K]
	A0A0H3GDX6		A0A0H3GDX6 [60-73]	[D].YAKDAPKLTDSKLR.[D]
	A0A0H3GDX6		A0A0H3GDX6 [317-326]	[D].GLTIHTALDR.[D]

*Lm* 10403S protein Name	*Lm* 10403S protein Accession number	Name of the homologous gene in *Lm* serovar 1/2a (strain ATCC BAA-679 / EGD-e)
Internalin D	A0A0H3G8X7	lmo0263
Flagellar hook capping protein	A0A0H3GA06	lmo0696
Peptidoglycan bound protein	A0A0H3GAK6	lmo0880
DUF5105 domain-containing protein	A0A0H3GAX9	lmo0791
Multiple sugar transport system substrate-binding protein	A0A0H3GCT3	lmo0181
Uncharacterized protein (PepSY domain-containing protein)	A0A0H3GCV0	lmo0047
1-phosphatidylinositol phosphodiesterase	A0A0H3GCV8	lmo0201
Invasion-associated protein p60	A0A0H3GDY4	lmo0582
Thioredoxin-like_fold domain-containing protein	A0A0H3GFB7	lmo1059
Phosphocarrier protein HPr	A0A0H3GFK1	lmo1002
Antigen A	A0A0H3GGY8	lmo0118
Beta-lactamase domain-containing protein	A0A0H3GI09	lmo0540
N-acetylmuramoyl-L-alanine amidase	A0A0H3GJB3	lmo2591
DNA-directed RNA polymerase subunit alpha	A0A0H3GJC8	lmo2606
Competence protein ComEA	A0A0H3GKB9	lmo1484
Uncharacterized protein	A0A0H3GMD3	lmo2203
Putative septation protein SpoVG	A0A0H3GD80	lmo0197
Peptide/nickel transport system substrate-binding protein	A0A0H3GH20	lmo0152
Penicillin binding protein 2B	A0A0H3GLV8	lmo2039
FeS assembly ATPase SufC	A0A0H3GMZ0	lmo2415

Cell-free *Lm* 10403S supernatants were treated with purified caspase-3 before analysis by TAILS degradomics (upper part) and by comparative 2D SDS-PAGE (lower part). The 9 overlapping proteins in the two approaches are indicated at the top of the protein list. In the TAILS proteomics list the identified cleavage site are shown on the right. Only proteins are listed that were found in three independent replicates of the proteomics assays.

For the virulence mediators LLO and the invasion associated protein p60 (Iap), an extracellular endopeptidase [4], the proteomics data were experimentally validated. Caspase-3 efficiently and dose-dependently cleaved LLO in *Lm* supernatants (Fig. 5G), as well as LLO and Iap as purified proteins (Fig. 5H). The detected cleavage fragments of LLO (c-term 52 kDa) and Iap (c-term 45.1 kDa) correspond to the top score sites according to SitePrediction [35] (Figure S3A), as well as to ScreenCap3 [36].

The detection of secreted *Lm* proteins in infected host cells is highly challenging as miniscule amounts of bacterial proteins are mixed with overwhelming amounts of host proteins. However, we were able to detect a band in the lysates of *Lm* infected HeLa cells that corresponds in size to the 13 kDa cleavage fragment of LLO mediated by caspase-7 (Figure S3B). This band is suppressed by DEVD-fmk and induced by TNF-α. However, as this is the only indication for intracellular *Lm* protein cleavage by caspase-3 at this point, and the blots needed to be overexposed to saturation (demonstrating various unspecific bands) to detect the potential cleavage fragment, we added this finding to the supplementary information. Further study is needed for firm conclusions.

### Caspase-uncleavable LLO or Iap mutants render *Lm* more virulent in HeLa cells

To test if executioner caspase-mediated degradation of LLO directly affects intracellular bacteria growth, we generated a *Listeria* line that secretes caspases-3 and -7-uncleavable, recombinant LLO in ΔLLO *Lm*. Cleavage SitePrediction software (and our caspase-3 TAILS proteomics data, see Table 1) predicted top score sites at the aspartate positions D62 (caspase-3) and D416 (caspase-7) in the LLO sequence (Figure S3A).

**Fig. 6:**
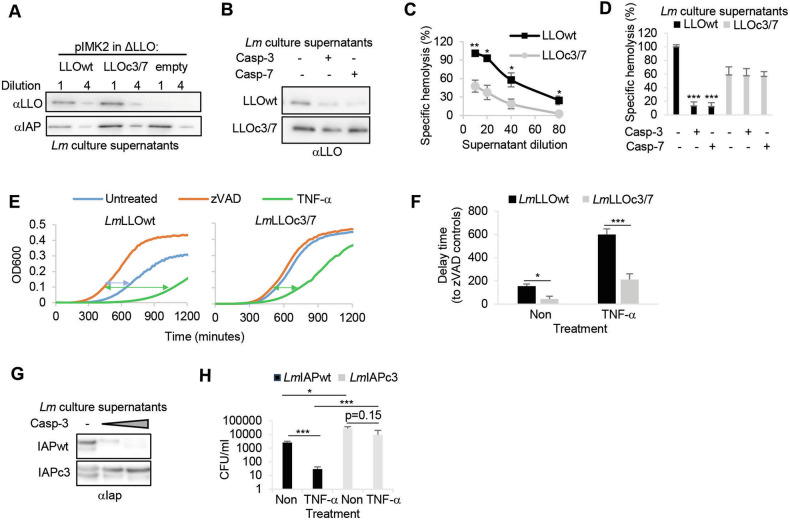
A *Lm* lines secreting caspases-3 and -7-uncleavable LLO or caspase-3-uncleavable Iap are more virulent in HeLa cells. Culture supernatants of ΔLLO *Lm*, transfected with indicated pIMK2-LLO constructs (**A**), were treated with caspase-3 or caspase-7 for 4 h (**B**). Secretion and caspase-mediated cleavage of LLO were assessed by immunoblots. Detection of Iap served as loading control in (**A**). **C** Human red blood cells were treated with serial dilutions (**C**) or caspase-treated (**D**) *Lm* supernatants containing LLOwt or LLOc3/7 for 15 min at 37 °C. Hemoglobin release was measured spectrophotometrically at 405 nm wavelength and average +/− SEM of three independent experiments is presented. **E** HeLa cells were infected with indicated *Lm* mutants +/− zVAD +/− TNF-α treatment before the intracellular bacterial load was assessed by growth curves assay. Arrows indicate the differences in delay times to the zVAD controls to reach the threshold OD of 0.1 that is plotted in (**F**). Average +/− SEM of the delay time normalized to zVAD controls in three independent experiments is shown. **G** *Lm* culture supernatants of lines secreting caspase-3-uncleavable (IAPc3) or wild-type Iap (IAPwt) were treated with caspase-3 for 4 h and cleavage of Iap was assessed by immunoblot. **H** HeLa cells were infected with indicated *Lm* mutants +/− TNF-α before the intracellular CFU were calculated from growth curves. Average +/− SEM of three independent experiments is shown. Significant differences between indicated groups are marked by asterisks. *P*-values are * < 0.05, **< 0.01 and ***< 0.005.

Comparison with the LLO structure revealed that these sites are well accessible as potential protease targets [37] (Figure S3C). Therefore, we replaced these potential cleavage site aspartates with glutamates and integrated the mutated LLO into the chromosome of ΔLLO *Lm* to generate *Lm*LLOc3/7 (Fig. 6A). The treatment of supernatants from *Lm*LLOwt and *Lm*LLOc3/7 with caspases-3 or -7 confirmed protection of the mutated protein (Fig. 6B). As the D62 to E point mutation is within the PEST domain critical for activity [38], we tested the hemolytic activity in the supernatants. The activity of the mutated LLO was indeed significantly decreased (Fig. 6C). However, contrary to the LLOwt supernatants, neither caspase-3 nor caspase-7 affected the hemolytic activity of LLOc3/7 (Fig. 6D). Due to this difference in the hemolytic activities, we could not directly compare the virulent growth of these two lines in host cells. To circumvent this difficulty, we compared the growth upon treatment with zVAD where all caspase activity is blocked (Fig. 6E). Indeed, in the LLOc3/7 mutant strain, the time differences to the zVAD controls in untreated and particularly in TNF-α treated conditions were significantly reduced as compared to LLOwt *Lm*, indicating a growth advantage mediated by caspase-uncleavable LLO (Fig. 6F). As Iap is highly expressed in *Lm* supernatants and efficiently cleaved by caspase-3 (Fig. 5H), but not caspase-7 (not shown), we additionally mutated the cleavage site aspartate D48 (to glutamate) in Iap to generate a caspase-3-uncleavable mutant (Fig. 6G). This mutant was hemolytic unimpaired (not shown) and the mutation is far away from the c-terminal catalytic domain NlpC/P60 [39], allowing direct comparison of the wild type and the mutant line. Growth assays in HeLa cells revealed a significant growth advantage of the mutant compared to wild-type *Lm*, which was again amplified by the presence of TNF-α (Fig. 6H). Overall, these findings directly link the caspase-mediated degradation of virulence mediators to intracellular *Lm* growth.

## Discussion

In this study, we present compelling evidence for an underappreciated host defense mechanism against intracellular *Lm* that is mediated by executioner caspases-3 and -7. Executioner caspase activity is robustly activated in HeLa cells upon infection with virulent *Lm*, presumably via the intrinsic pathway and the activation of caspase-9. By engaging the extrinsic pathway and triggering caspase-8 activation, downstream caspase activity can be significantly enhanced by simultaneous TNF-α treatment. It was reported earlier that *Lm* infection triggers nucleosomal DNA fragmentation in mouse hepatocytes [40], presumably mediated by caspase-3 [41]. Caspase-7 activation upon *Lm* infection in murine macrophage was demonstrated to exert a cytoprotective effect on the host cells [42]. In this study, caspase activation was shown to not require caspase-1 or key innate signaling molecules, such as ASC, RIP2 or MyD88. However, how exactly *Lm* infections trigger executioner caspase activity remains elusive.

Though remarkably high cytoplasmic caspase-3 and/or -7 activity was recorded in HeLa cells upon *Lm* infection, particularly in presence of TNF-α, the cells stayed viable according to nuclear morphology, cytoskeleton organization, plasma membrane integrity and mitochondrial metabolization rate, at least at the low multiplicities of infection (MOI ≤ 1) used in this study. At this point, we can only speculate why activated executioner caspases upon *Lm* infections (and caspase-9 activation) did not translocate into the nucleus to further proceed with the host cell death program by degrading nuclear substrates [26]. The executioner caspases may be sequestered in the cytosol by the increased supply of protein substrates secreted from *Lm*, as suggested by our identification of a 13 kDa LLO fragment, presumably provided by caspase-7 proteolytic activity in the cytosol of infected host cells. However, at higher MOI ≥ 10, infected HeLa cells die rapidly, demonstrating signs of multiple death programs as this was recently observed and named PANoptosis [43], potentially indicating that at higher infection doses this cellular immune defense mechanism might be overwhelmed and additional inflammatory programs are need to fight the infection.

More importantly, we additionally demonstrate that the chemical inhibition of DEVDase activity or the genetic depletion of both caspases-3 and -7 decreases the resistance of HeLa cells against intracellular *Lm*. Surprisingly, caspase depletion did not affect resistance to *Salmonella Typhimurium* that highly induces executioner caspase activity, and crucial virulence effectors (SipA, SifA) are degraded by active caspase-3 [21, 44]. However, in contrast to *Lm*, effector cleavage might be even beneficial for the dissemination of *Salmonella*, suggesting major species-specific differences in caspase-mediated anti-bacterial defense [21]. The beneficial caspase-3 cleavage of SipA for the dissemination of *Salmonella* was explored predominantly in an experimental in vivo model, highlighting the need of more comprehensive in vivo studies to define the role of executioner caspases in antibacterial immunity.

Interestingly, in the monocytic acute leukemia line, THP-1, the single knock-out of caspase-7 led to increased intracellular bacteria growth of both *Lm* and *Salmonella*. Caspase-7, though structurally closely related to caspase-3, has a dual role in cell death and inflammation. Unlike caspase-3, it is activated by inflammatory processes, including active caspase-1 [45]. Active caspase-7, independently of caspase-1, was detected upon *Lm* infection [42], as well as during intracellular *Salmonella* and *Legionella pneumophila* infections, downstream of caspase-1 [46–48]. Remarkably, caspase-7-deficient mice allowed increased growth of *L. pneumophila* in their macrophages in vitro, and in their lungs in vivo [49]. Substantial replication of *L. pneumophila* was also observed in dendritic cells of caspase-3-deficient mice [50], suggesting that some gram- bacteria are also susceptible to executioner caspase activity. Using the unique model of gut specific caspase deletion, it was recently demonstrated that caspase-3 and -7 are required in intestinal epithelial cells to restrict Clostridium difficile infections [51], again highlighting the need for additional in vivo studies to clarify the role of executioner caspases during bacterial infections.

A crucial role of caspases-3 and -7 in immune defense against *Lm* was additionally demonstrated by the increased growth of a mutant strains that secretes caspase-uncleavable LLO or Iap in HeLa cells. Both LLO and Iap are also substrates of human granzyme B [14]. This overlap in substrate selection by granzyme B in general is not uncommon, as it shares cleavage specificity after aspartate residues with the caspases [52] and activates numerous caspases by direct cleavage, including caspase-3 [53] and caspase-7 [54]. To induce apoptosis, granzyme B can directly process numerous caspase substrates, such as Parp1, NuMA, DNA-PK or ICAD [55, 56].

Comprehensive proteomics analysis of caspase-3 substrates in the *Lm* secretome identified proteins critically involved in pathogen-host interactions and virulence. The top enriched pathways include membrane transport, in particular via the ATP-binding cassette (ABC) transporter complex [57], the PrfA-dependent virulence factors LLO, ActA, PlcA and PlcB [58], peptidoglycan catabolic hydrolases, such as Iap, and proteins anchored to the outer surface via Gly-Trp (GW)-domains, including InlB [59], all pathways critical for full *Lm* virulence in a host. The pathways and the overall protein substrate network is remarkably interconnected with highly significantly enriched interactions, indicating a targeted attack of caspase-3 on proteins that are essential for pathogen-host interactions. The top subcellular localization was as expected the extracellular region. However, the screen revealed in addition a multitude of membrane and cytoplasmic proteins. A potential interpretation of the presence of these types of proteins in cell-free supernatants is provided by the comparison of the caspase-3 substrate list with a recent independent proteomics analysis of *Lm* membrane vesicles [60]. 93.1% of the caspase-3 substrates were also found with high confidence (in three replicate analyses) in highly purified membrane vesicles. To conclude how the release of membrane vesicles mediates *Lm* virulence and how the caspases interfere with it needs extensive further study.

In conclusion, this study identifies the executioner caspases-3 and -7 as a novel innate immune barrier against intracellular growing *Lm*. This barrier is established by the targeted degradation of a multitude of bacterial proteins that are critically involved in pathogen-host interactions, therefore inhibiting virulent growth.

## Methods and Materials

### Human cells and cell culture conditions

HeLa cells (Abcam, *Mycoplasma* free) were cultured in DMEM (Pan Biotech, P04-04510), supplemented with 10% heat-inactivated FBS (Sigma) and 1% antibiotic/antimycotic solution (Thermo Fisher).

THP-1 cells (Sigma, *Mycoplasma* free) were cultured in RPMI-1640 (Pan Biotech, P04-18500), supplemented with 10% heat-inactivated FBS (Sigma), 50 μM 2-mercaptoethanol (Sigma), and 1% antibiotic/antimycotic solution (Thermo Fisher).

### Bacterial Strains

*Listeria monocytogenes* 10403S and 10403S ΔLLO, EGD-e and EGD-e ΔIap, and *Salmonella enterica* serovar Typhimurium SL1344 used for infections were grown to mid-log in appropriate medium (Brain Heart Infusion (Millipore, 53286) + 50 μg/ml streptomycin for 10403S and 5 μg/ml erythromycin for EGD-e; Luria broth (Sigma, L3022) + 50 μg/ml streptomycin for *Salmonella*). 50 μg/ml kanamycin was added to grow mutant *Listeria* (pIMK2 transfected) [61]).

### Gene modification by CRISPR/Cas9 methodology and clone selection limiting dilution

The gene editing was based on the nucleofection (4D Nucleofector System, Lonza) of preformed Cas9-guideRNA-ribonucleoprotein (RNP) complexes into target cell line (HeLa, THP-1) according to manufacturer’s recommendations (IDT). Three guides per gene were tested and the efficiency of knockdown was assessed by western blot. The cells that displayed most efficient knockdown (usually around 50%) were used for downstream dilution assays. HeLa cell gene edits were started using a commercial caspase-3 KO and the corresponding parental lines (abcam, ab255370, ab255448). Most efficient guide sequences were GATCGTTGTAGAAGTCTAAC for caspase-3 (in THP-1 cells) and GATATGTAGGCACTCGGTCC for caspase-7. Monoclonal cell populations were selected by seeding in an average of 0.5 cells in 100 μl in 96-well plates for 7 days before subcloning in repeated standard limiting dilution assays.

### Generation of a caspase-uncleavable LLO and Iap mutant

Full-length (including the natural ribosome binding site and signal peptide) LLO and Iap were PCR amplified from the chromosome of *Lm* 10403S and EGD-e, respectively. The amplicons were cloned into the bacterial expression vector pGEX4Ti (Sigma) using the BamH1 and Xho1 restriction sites. Cleavage sites for caspases were predicted using the SitePrediction software [35] and experimentally validated. The validated top score cleavage site aspartic acids were mutated to glutamic acid by sequential two-step overlap PCRs [62]. Correct point mutations were confirmed by sequencing (Microsynth AG, Balgach, Switzerland). Wild-type and mutated genes were cloned into the integration vector pIMK2 [61] at the BamH1/Xho1 sites, electroporated into LLO- or Iap-deficient *Lm* (10403S and EGD-e, respectively). The mutants were selected on kanamycin BHI agar plates to generate the lines *Lm*LLOwt and *Lm*LLOc3/7, as well as *Lm*IAPwt and *Lm*IAPc3.

### Hemolysis assays

Serial dilutions (10-80fold) of *Lm* culture supernatant were incubated with human red blood cells at a hematocrit of 0.4% in hemolysis buffer (100 mM NaCl, 40 mM NaPO_4_, 0.5 mg/ml BSA, pH=5.5) in u-bottomed microtiter plates at 37 °C for 15 min. After the incubation, the plate was spun (500 x g, 3 min) and the supernatant was transferred to a flat-bottomed microtiter plate. Hemolysis was assessed by absorbance readings at 405 nm in a plate reader (Synergy H1, Biotek). Specific hemolysis was normalized to positive control lysis induced by 0.1% Triton X-100, corrected by the spontaneous hemoglobin release in buffer only conditions. For some experiments, a 10fold dilutions of the *Lm* culture supernatants were pretreated with 2 U/μl of purified caspase-3 (see below) or commercial caspase-7 (Enzo Life Sciences) for 4 h at 37 °C before the assessment of the hemolytic activity.

### Bacterial infections, colony forming unit (CFU), growth assays, DEVDase activity assessment and caspase activation

Before infections, overnight cultures of bacteria were diluted 1:50 in fresh broth and grown to mid-log, then were washed with PBS and resuspended in infection medium (RPMI + 1% BSA + appropriate antibiotics as above). Cell density was estimated by OD600 spectrometry (OD_600_ = 0.1 corresponds to ~2 × 10^7^ bacteria/ml) and confirmed by CFU assay.

HeLa and THP-1 cells were infected with *Lm* 10403S and *Salmonella enterica* serovar Typhimurium SL1344 for 60 min at indicated multiplicity of infection (MOI) in 24-well plates in triplicates. The infected cells were washed throuroughly with PBS and then further incubated with gentamicin (25 μg/ml) in infection medium. For some experiments, particularly when using higher MOIs due to cytopathic effects and subsequent susceptibility to gentamicin, gentamicin treatment was only for 30 min, followed by further incubation in gentamicin-free infection medium that was exchanged every 4 h. In some experiments, 20 μM zVAD, 20 μM zDVED-fmk or 10 ng/ml TNF-α was added. At indicated times, samples were washed with PBS and then hypotonically lysed by adding ice-cold sterile water for 45 min on ice.

For CFU assays, lysates were serially diluted in broth and spread on LB-Agar plates containing the appropriate antibiotics. Colonies were enumerated after 24 h at 37 °C.

For the growth assays, lysates were 10fold diluted in flat-bottomed 96-well plates and the OD at wavelength 600 nm was measured every 15 min while discontinuous shaking in heat-controlled plate reader for 24 h at 37 °C (Synergy H1, BioTek).

For the colorimetric DEVDase activity measurement (only in HeLa cells), lysates were cleared by centrifugation, and the supernatants were 10fold diluted into caspase assay buffer (50 mM Tris, pH 7.5, 0.3% NP-40, 1 mM DTT) containing 200 μM Ac-DEVD-pNA (Sigma). Cleavage was monitored colorimetrically at 405 nm after 4 h at 37 °C. Due to general lower DEVDase activity in THP-1 cells, DEVDase activity was measured fluorometrically. For this purpose, TF3-DEVD-FMK (Cell Meter™ Live Cell Caspase-3/7 Binding Assay Kit, AAT Bioquest) at 1:150 ratio was added to the cells 60 min before the experimental endpoint. Cells were washed twice for 3 min in Washing Buffer (Kit component B) before fluorescent intensity was monitored in the well area scanning mode at Ex/Em = 550/595 nm in the Synergy H1 plate reader.

Caspase activation in the lysates was directly detected by western blot using antibodies against active caspases-3, -7 and -9, and cleaved Parp1 (Cleaved Caspase Antibody Sampler Kit #9929, Cell Signaling), as well as against caspase-8 (Initiator Caspases Antibody Sampler Kit #12675, Cell Signaling) according to manufacturer’s recommendations.

### Assessment of host cell viability by MTS assay, LDH release and microscopy

2 h before the experimental endpoint, MTS reagent (MTS Assays Kit, abcam) was added (1:10 ratio) to host cells (treated as above), the absorbance was then measured at 490 nm wavelength.

Cells were gently spun (300 x g, 3 min) before the supernatant was transferred into flat-bottomed 96-well plates for the assessment of LDH release (Cytotoxicity Detection kit, Roche) according to the manufacturer’s recommendations. To some wells, Triton X-100 (Sigma) was added to a final concentration of 0.1% before the centrifugation to determine the maximal release.

For microscopy, HeLa cells were seeded in culture medium at a density of 10^5^ cells in 200 μl on glass coverslips in 24-well plates overnight, and then infected and treated in infection medium with *Listeria* as above. 1 h before fixation, FITC-DEVD-fmk (abcam) was added to the cells to a final concentration of 60 μM. The cells were then fixed and washed twice with PBS before staining with phalloidin-AF647 (250 nM, ThermoFisher) and Hoechst (1 μg/ml, Sigma) for 30 min at room temperature in the dark.

Additionally, HeLa cells were infected and treated as above with *Lm*, prestained with 2 μM CFSE (Sigma) for 30 min on ice. To assess early cell death, infected cells were fixed in cold methanol (−20 °C) for 15 min, washed twice with PBS and then stained with the CytoDEATH M30 antibody (Roche) and Hoechst (1 μg/ml, Sigma) for 1 h at room temperature in the dark. After the primary antibody, cells were washed with PBS and then counterstained with anti-mouse IgG-AF594 (R&D Systems) for 30 min at room temperature.

As positive control to induce cell death in these experiments, some wells were treated with 0.1 μg/ml staurosporine (STS).

All stained cover slips were washed twice with PBS before mounting in Vectashield (Vectorlabs) and analysis by confocal microscopy (Leica SP5).

### Caspase-3 purification

Recombinant, human caspase-3 was purified from *E. coli* as described [63, 64]. In brief, the pET21b-Caspase-3 plasmid (Addgene) was transformed into BL-21 *E. coli*. These cells were grown to a density of A_600nm_ = 0.6–0.8 at 37 °C and 220 rpm in 500 ml of induction medium (20 g/l Tryptone, 10 g/l yeast extract, 5 g/l NaCl, 0.4% glucose, 1 mM MgCl_2_, 0.1 mM CaCl2) containing 0.1 mg/ml ampicillin. Isopropyl-1-thio-b-D-galactopyranoside (IPTG, 1 mM) was added, and the culture was shaken at 25 °C, 200 rpm for 3 h. Cells were pelleted (centrifugation 3000 x g for 12 min) and resuspended in 50 ml of His binding buffer (100 mM Tris-HCl, 20 mM imidazole, and 500 mM NaCl, pH 8.0) containing 0.1 mg/ml lysozyme and 0.1% Triton X-100. The cells are incubated for 40 min on ice and vortexed every 10 min. Then, the cells underwent three freeze-thaw cycles and a sonication to make the sample less viscous. After centrifugation (17’000 x g for 47 min at 4 °C), the supernatant was harvested and 50 ml of His binding buffer were added to dilute it. After filtration with a 0.22 µm filter, the supernatant was loaded onto a 5 ml HisTrap HP column (Cytiva, 17524801) equilibrated with His binding buffer. The purified caspase-3 protein was eluted from the column using a linear imidazole gradient (until 1 M imidazole). A sample of each fraction were used for a gel electrophoresis and Coomassie staining to select the fraction containing the caspase-3 protein. These fractions were mixed and concentrated using a 3 kDa MWCO Amicon filter (Millipore, UFC9003), and the buffer was changed by caspase-3 buffer (50 mM HEPES, pH 7.4, 0.1% CHAPS, 10 mM DTT, 100 mM NaCl, 1 mM EDTA and 10% sucrose).

### Assessment of caspase-3 substrate cleavage in the *Lm* secretome by comparative 2D SDS-PAGE and TAILS proteomics

*Lm* were grown to mid-log in 100 ml of BHI medium supplemented with 50 μg/ml streptomycin. Then, the bacteria were grown in 100 ml of RPMI-1640 medium (Pan Biotech, P04-18500) supplemented with 50 μg/ml streptomycin for 4 h at 37 °C at 180 rpm. The supernatant was harvested after centrifugation of the bacterial culture (4000 rpm for 15 min) and filtered with a 0.22 µm filter. The supernatant proteins were concentrated by ultrafiltration using a 3 kDa MWCO Amicon filter (Millipore, UFC9003), and the RPMI was exchanged by caspase-3 assay buffer (20 mM HEPES, pH 7.4, 0.1% CHAPS, 5 mM DTT, 2 mM EDTA). 50 µg of supernatant proteins were treated or not with caspase-3 (weight ratio caspase-3 to bacterial proteins 1/10) for 24 h at 37 °C and then precipitated by trichloroacetic acid precipitation. The samples were used either for 2D SDS-PAGE or TAILS proteomics assays.

For 2D SDS-PAGE, the precipitated proteins were resuspended into 300 µl of 2-D sample solution (7 M urea, 2 M thiourea, 4% (w/v) CHAPS, 40 mM DTT, 0.2% (w/v) Bio-Lyte® ampholytes pH3-10) and passively loaded into a 17 cm immobilized pH gradient (IPG) strip pH3-10 for 16 h (Bio-rad, 1632007). The proteins were then separated according to their isoelectric pH by isoelectric focusing. Thereafter, the IPG strip was treated with 1% w/v dithiothreitol (DTT) and 4% w/v iodoacetamide (IAA) for reduction and alkylation of proteins respectively. The proteins were then separated according to their molecular weight by electrophoresis. For this, the strip was placed on the top of a 12% polyacrylamide gel and fixed with 0.5% agarose solution. The 2D SDS-PAGE experiments have been carried out with the Bio-rad materials according to the provided instructions. For the visualization of protein spots, the gel was first fixed and then stained in silver stain (Silver stain plus kit, Bio-Rad, 1610449). Pictures of the stained gels were taken with the Perfection V850 Pro scanner (Epson). The Delta2D (DECODON) software was used to analyze the gel pictures and select the spots to pick up for mass spectrometry (MS) analysis. Spots whose intensities changed by at least a factor 2 upon caspase-3 treatment in three replicate analyses were selected for MS analysis. Before MS analysis, each spot was destained and the proteins were digested by trypsin, extracted from the gel pieces, and cleaned up.

For the TAILS, a protocol adapted from Kleifeld et al. [29] was used. Briefly, the precipitated proteins were resuspended into 50 µl of TAILS buffer (2.5 M GuHCl, 250 mM HEPES, pH 7.8). The proteins were denaturated with 1 mM Tris(2-carboxyethyl)phosphine hydrochloride (TCEP) for 1 h at 65 °C, and alkylated with 5 mM chloroacetamide (CAA) for 30 min at 65 °C. The N-termini were labelled with stable isotopes (TMTsixplex™ Isobaric Label Reagent Set, ThermoFisher, 90061) for 1 h at room temperature. The quench labelling reaction was then done with a final concentration of 100 mM ammonium bicarbonate (NH_4_HCO_3_), for 30 min at room temperature. The clean-up of samples was performed by the addition of ice-cold acetone (7 volumes) and methanol (1 volume), followed by the incubation of samples for 2 h at −80 °C. After centrifugation at 4’700 rpm for 20 min, the protein pellet was washed with 5 mL of ice-cold methanol and then resolubilized with 100 mM NaOH solution (as little as possible), followed by the addition of HEPES buffer, pH 7.8, to a final concentration of 100 mM. Trypsin (Promega, V5113) was added at a 1:100 ratio (enzyme/substrate), and the mixture was incubated at 37 °C for 18 h. Adjust the pH of the samples to pH 6-7 using 2 M HCl. Add fivefold excess (w/w) of hyperbranched polyglycerol-aldehydes (HPG-ALD) polymer (Flintbox) and 5 M NaBH_3_CN to a final concentration of 50 mM NaBH_3_CN and incubate at least 16 h at 37 °C. Thereafter, the polymer is separated from the unbounded peptides by ultrafiltration using a 30 kDa MWCO Amicon filter (Millipore, UFC5030). The TAILS samples were acidified to pH < 2 using 10% trifluoroacetic acid (TFA) and cleaned up. For this, the proteins were loaded onto a column made of C18 solid phase extraction (SPE) disks (Empore, 66883-u). The samples were washed twice with 0.1% formic acid, eluted with a solution of 80% acetonitrile, 0.1% TFA, and completely dried under vacuum.

### Mass spectrometry analysis and data extraction

Liquid Chromatography Mass Spectrometry/ Mass Spectrometry (LC-MS/MS) measurements were performed on a Q Exactive HF-X mass spectrometer (Thermo Scientific) coupled to an EASY-nLC 1000 nanoflow-HPLC (Thermo Scientific). Peptides were separated on a fused silica HPLC-column tip (75 µm inner diameter (New Objective), self-packed with ReproSil-Pur 120 C18-AQ, 1.9 µm particle size (Dr. Maisch GmbH) to a length of 20 cm) using a gradient of A (0.1% formic acid in H_2_O) and B (0.1% formic acid in 80% acetonitrile in H_2_O): samples were loaded with 0% B with a flow rate of 600 nL/min; peptides were separated by 5–30% B within 85 min with a flow rate of 250 nL/min. Spray voltage was set to 2.3 kV and the ion-transfer tube temperature to 250 °C; no sheath and auxiliary gas were used. The mass spectrometer was operated in the data-dependent mode; after each MS scan (mass range *m/z* = 370–1750; resolution: 120,000), a maximum of twelve MS/MS scans were performed using an isolation window of 1.6, a normalized collision energy of 28%, a target Automatic Gain Control of 1e5 and a resolution of 30,000. MS raw files were analyzed with the MaxQuant software [65], using the UniProt full-length *Listeria monocytogenes* proteome (UP000001288), additionally including common contaminants (e.g., keratin) and trypsin, as reference. Carbamidomethylcysteine was set as fixed modification and protein amino-terminal acetylation and oxidation of methionine were set as variable modifications. The MS/MS tolerance was set to 20 ppm and three missed cleavages were allowed using Trypsin/P as enzyme specificity. Peptide and protein false discovery rates (FDR), based on a forward-reverse database, were set to 0.01, minimum peptide length was set to 7, and minimum number of unique peptides for identification of proteins was set to one. The “match-between-run” option was used with a time window of 0.7 min. MS raw files of TAILS experiment were processed using Proteome Discoverer software (Thermo Scientific) following the protocol of Madzharova et al. [66].

### Experimental validation of the proteomics data

Cleavage of native LLO was experimentally confirmed by treating cell free *Lm* culture supernatant (as above) with indicated concentrations of purified caspase-3 at 37 °C and analyzed by immunoblot using rabbit anti-LLO antibodies (Abcam).

In addition, LLO-GST and Iap-GST fusion proteins using the constructs, pGEX4Ti-LLO or pGEX4Ti-Iap, respectively, in *E. coli* BL21 were purified on a GST column (GSTtrap HP, GE Healthcare) following the manufacture’s recommendation. These fusion proteins were treated with indicated concentrations of caspase-3 for 4 h and analyzed on Coomassie stained SDS-PAGE.

### Statistics

All experiments were performed in triplicates and were repeated at least three times independently. Data are presented as means ± SEM. Comparisons between the different groups with similar variance were performed with two-tailed unpaired Student’s t-tests (using Microsoft Excel). *P*-values of less than 0.05 were considered significant. For the growth experiments in Fig. 2C, D, G, H, significant differences refer to the measured raw data of lag times before calculation of CFUs.

## Supplementary information

Supplementary figure S1 to S3

Uncropped western blots

## Data Availability

The proteomics dataset generated and analyzed during in this study is available in Table 1. All data needed to evaluate the conclusions in the paper are present in the paper and/or the Supplementary Materials. All materials are available under request to the corresponding author.
