# Supplementary material for: Executioner caspases degrade essential mediators of pathogen-host interactions to inhibit growth of intracellular Listeria monocytogenes
Source: Cell Death Dis. 2025 Jan 30;16(1):55. doi: 10.1038/s41419-025-07365-x (PMC11782612; doi:10.1038/s41419-025-07365-x)
Supplement: Supplementary file 1 — Supplementary figure S1 to S3 [file 41419_2025_7365_MOESM1_ESM.pdf]

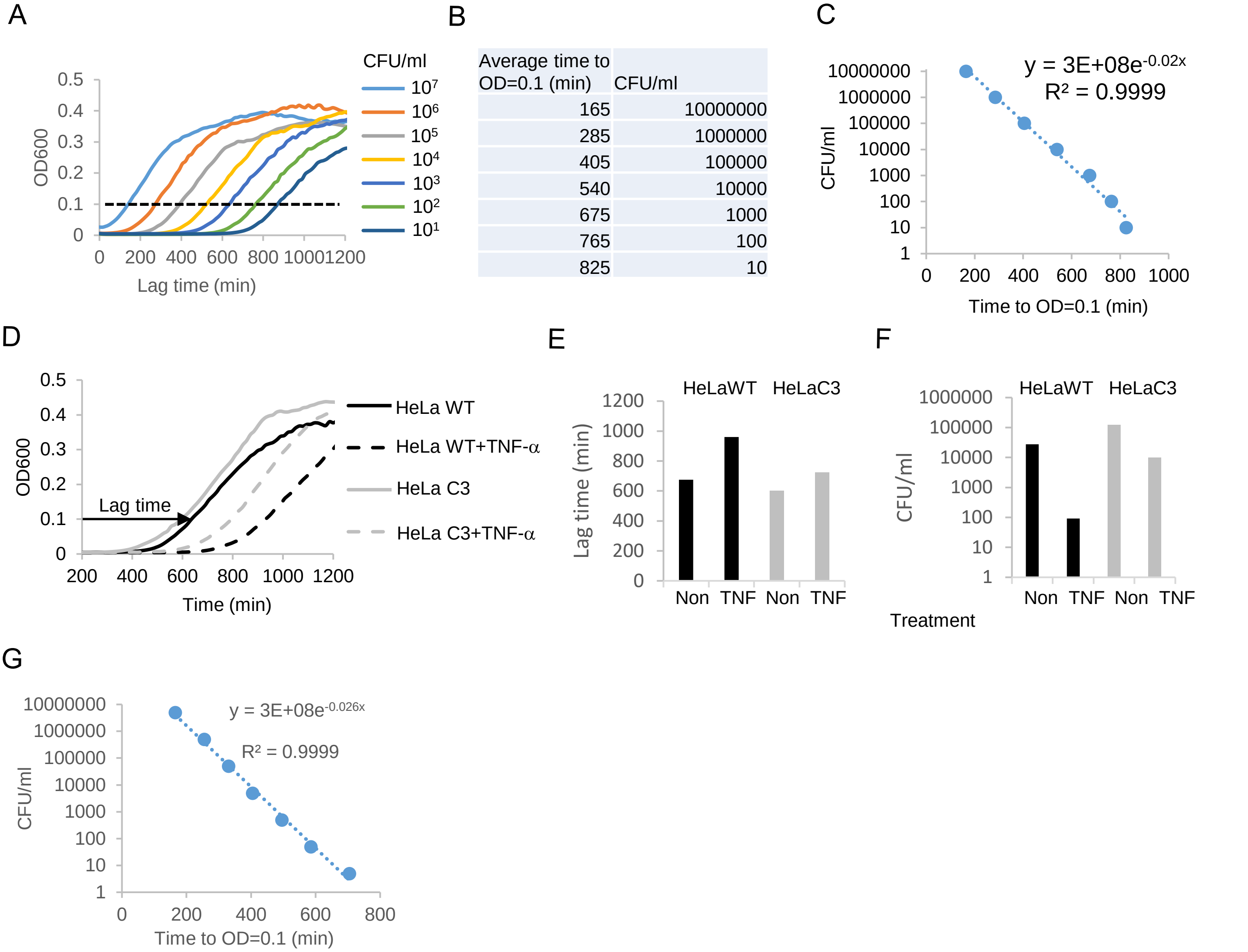

Average time to OD=0.1 (min)	CFU/ml
165	10000000
285	1000000
405	100000
540	10000
675	1000
765	100
825	10

Figure S1: Calculation of bacterial numbers from standard bacterial growth curves. *Lm* of known concentrations (due estimation according to OD600 and confirmation by CFU assays) were serially diluted in BHI broth containing 50 µg/ml streptomycin and bacterial growth was monitored by overnight kinetics analysis at OD600 in a heat-controlled plate reader. Representative standard dilution growth curves are shown in **A**. The lag times for the growth curves of individual *Lm* concentrations to reach a threshold OD of 0.1 are listed in **B** and plotted in **C**. A trendline with corresponding equation and correlation value is indicated.

Caspase-3 deficient (C3) and parental (WT) HeLa cells were infected with *Lm* at MOI 0.1 for 16 hours +/-TNF-α before the cells were lysed. Lysates were diluted 1:10 in BHI broth and bacterial growth was monitored as above. Representative growth curves are shown in **D**. The black arrow indicates the lag time of a particular growth curves to reach a threshold OD of 0.1. The lag times are blotted in **E** and the corresponding CFUs in **F**. **G** shows the CFU-lag time correlation, including trend line and equation for *Salmonella* Typhimurium SL1344.

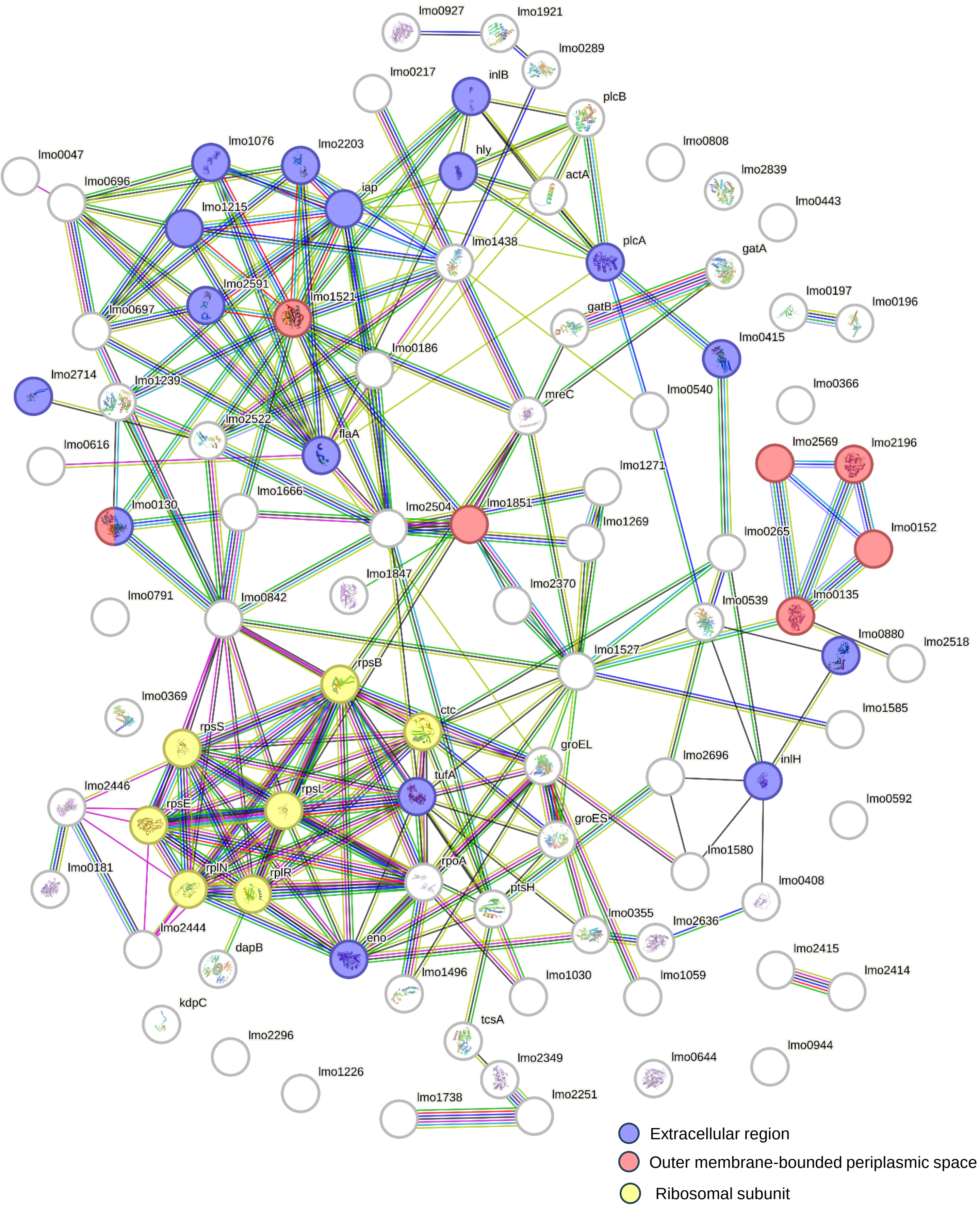

Figure S2: Protein-protein interaction (PPI) network analysis by STRING. Network nodes are 95 proteins of the input (108 proteins). Cellular components (extracellular, outer membrane bound, ribosomal) are indicated. Edges represent protein-protein associations. Number of edges is 231 with an expected value of 140, which indicates a PPI enrichment p-value of 1.08e-12.

A

Cleavage SitePrediction of LLO by human caspase-3

rank	position	site	N fragment	C fragment	frequency score	similarity maxscore	similarity maxsite	average score
1	59 to 64	DEID.KY	6.7 kD	52.0 kD	0.265	77.419	DEVDKM	20.496
2	413 to 418	AYTD.GK	45.6 kD	13.1 kD	0.308	64.516	AETDGQ	19.863
3	365 to 370	DLRD.IL	40.2 kD	18.4 kD	0.170	64.286	DVLDVL	10.912
4	317 to 322	AAFD.AA	35.3 kD	23.4 kD	0.056	63.333	TQFDAA	3.565

Cleavage SitePrediction of LLO by human caspase-7

rank	position	site	N fragment	C fragment	frequency score	similarity maxscore	similarity maxsite	average score
1	413 to 418	AYTD.GK	45.6 kD	13.1 kD	0.438	64.516	AETDGQ	28.286
2	317 to 322	AAFD.AA	35.3 kD	23.4 kD	0.029	53.125	SAFDGG	1.553
3	154 to 159	LSID.LP	17.2 kD	41.5 kD	0.027	53.333	VEVDAP	1.415
4	59 to 64	DEID.KY	6.7 kD	52.0 kD	0.014	63.333	DELDRS	0.894

Cleavage SitePrediction of Iap by human caspase-3

rank	position	site	N fragment	C fragment	frequency score	similarity maxscore	similarity maxsite	average score
1	45 to 50	TTVD.AI	4.6 kD	45.1 kD	0.132	60.714	TEVDAA	8.033
2	94 to 99	AGVD.NS	9.8 kD	39.9 kD	0.083	66.667	AAVDTS	5.536
3	124 to 129	TYND.GK	13.1 kD	36.6 kD	0.027	54.545	VYRDGT	1.493

B

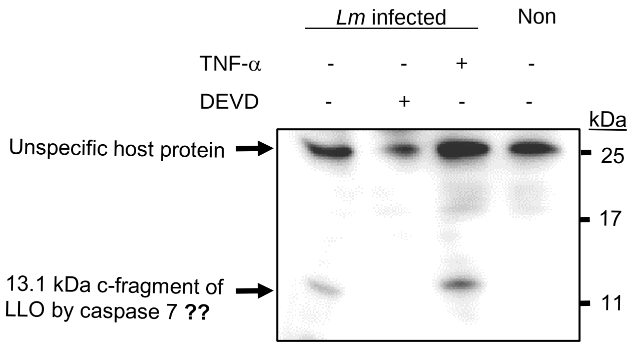

C

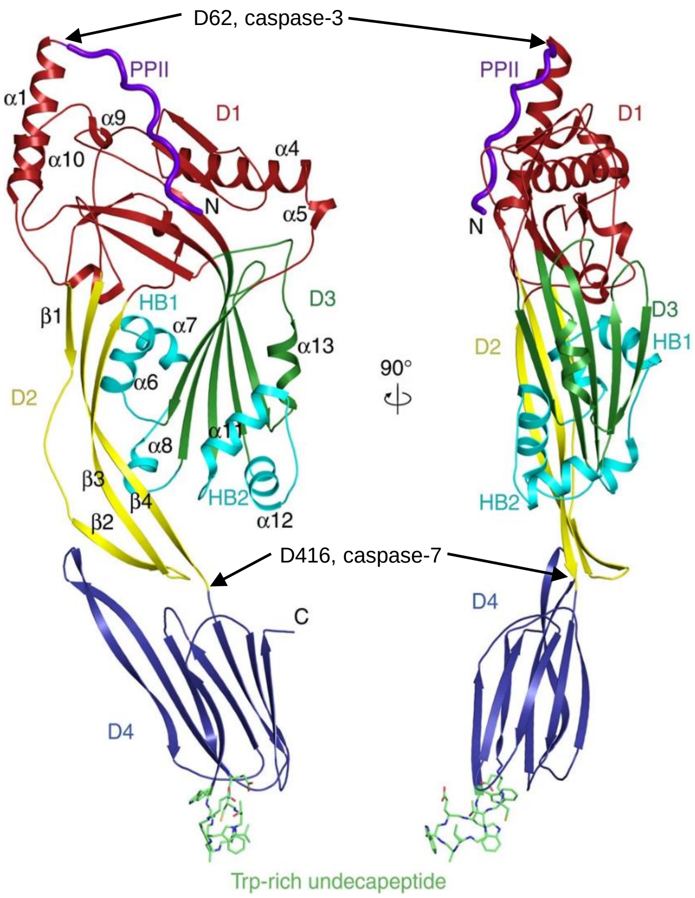

Figure S3: Cleavage SitePredition in LLO and Iap for human caspase-3 and -7, and 3D structure of LLO. (**A**) Cleavage sites for human caspases were predicted using the SitePrediction software (Verspurten, Gevaert et al. 2009) that relies on statistics calculations, and includes PEST sequences, solvent accessibility and secondary structure in the algorithm to predict cleavage sites. (**B**), HeLa cells were infected with *Lm* or not (Non) for 6 hours +/- TNF-α +/- DEVD-fmk before hypotonic lysis in ice–cold water. The lysates were spun (15`000 x g for 1 minute) to remove bacteria and the supernatants were assessed by LLO immunoblot. An unspecific host protein serves as loading control (**C**), LLO 3D structure according to (Koster, van Pee et al. 2014) is presented. The cleavages sites for caspases-3 and -7 are indicated by arrows.
