# Supplementary material for: Executioner caspases degrade essential mediators of pathogen-host interactions to inhibit growth of intracellular Listeria monocytogenes
Source: Cell Death Dis. 2025 Jan 30;16(1):55. doi: 10.1038/s41419-025-07365-x (PMC11782612; doi:10.1038/s41419-025-07365-x)

1B

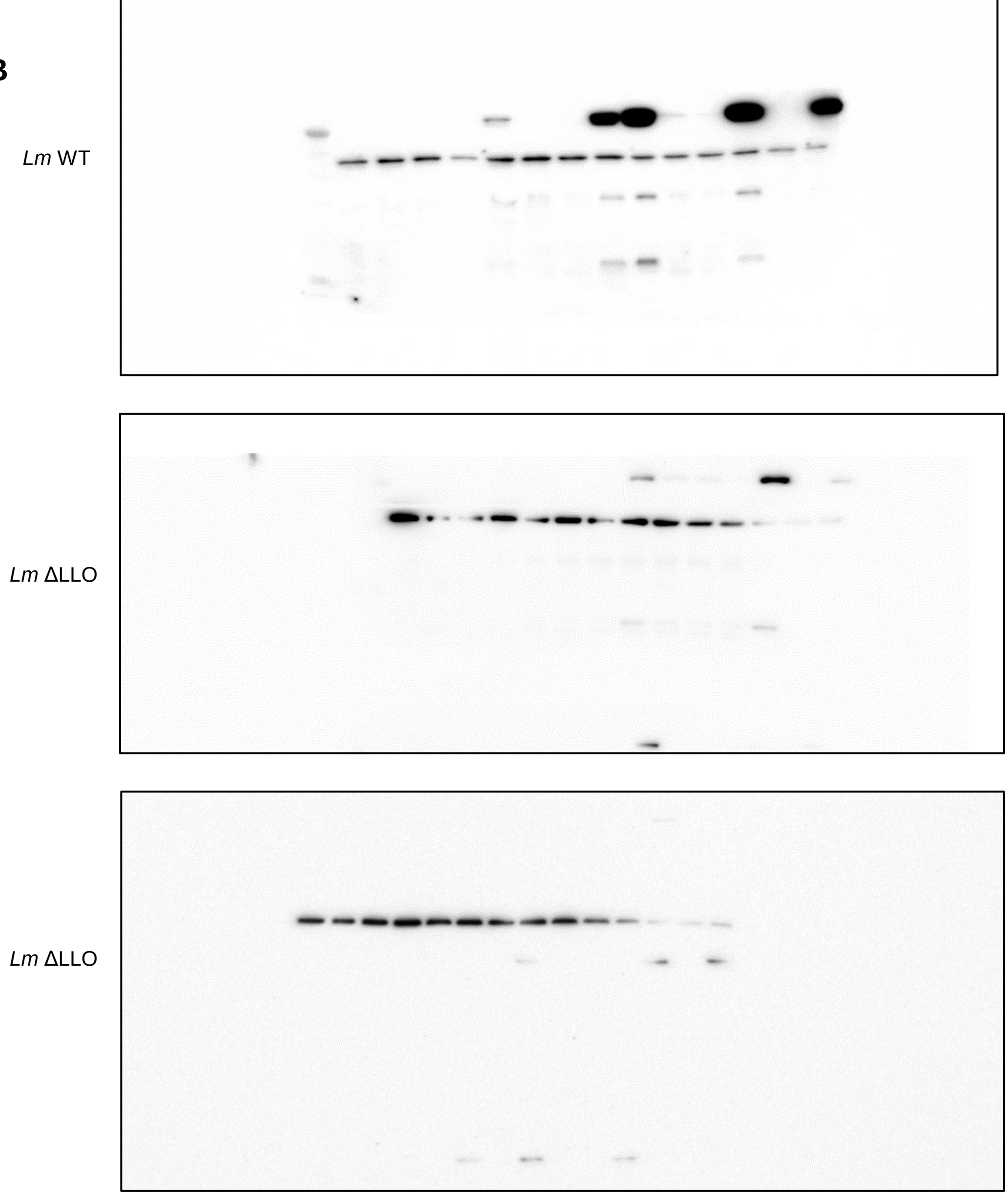

1B

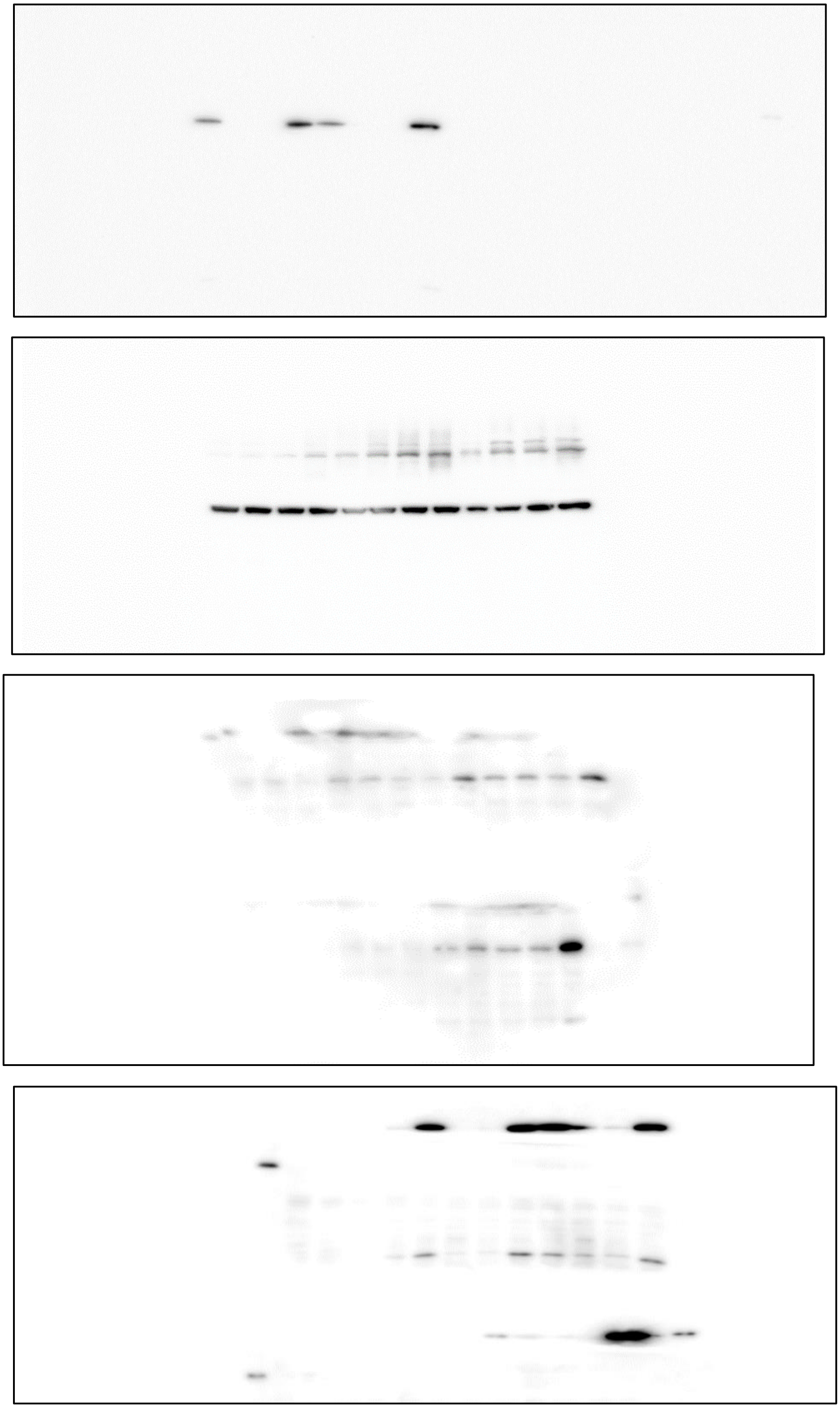

1C

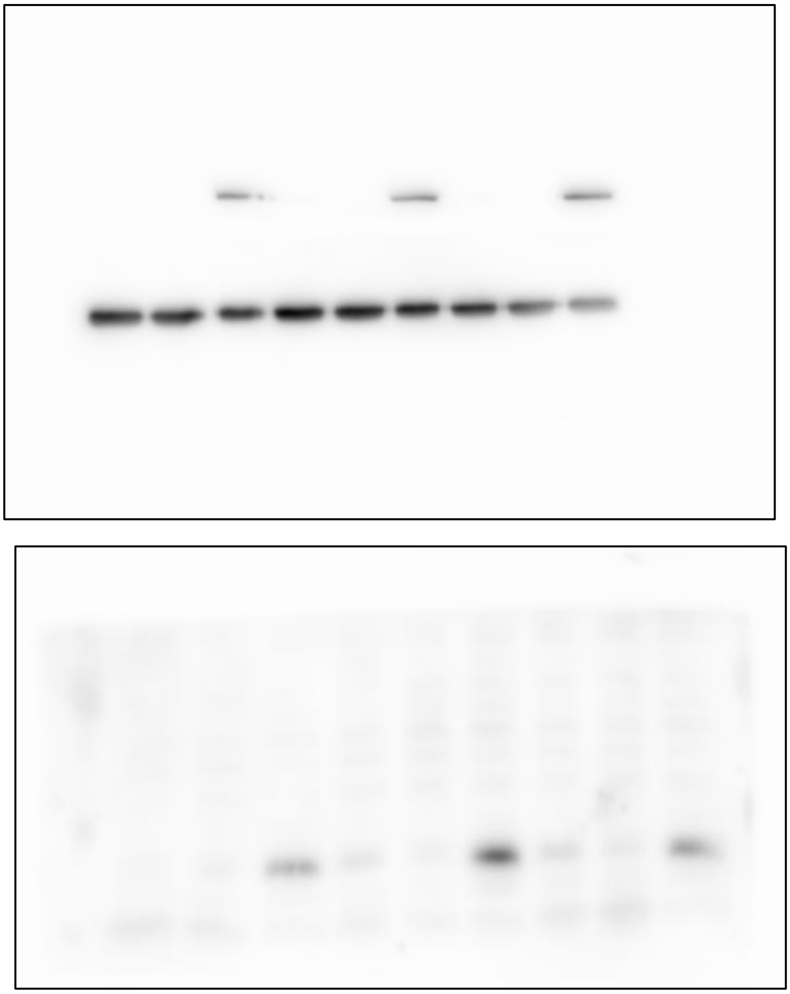

1D, E

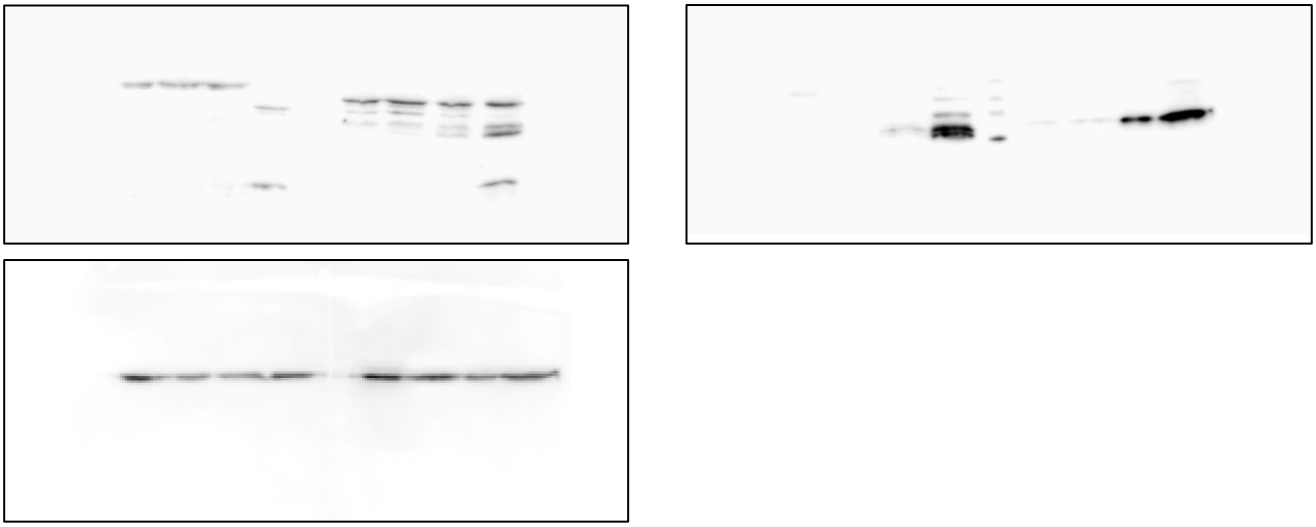

3A HeLa cells

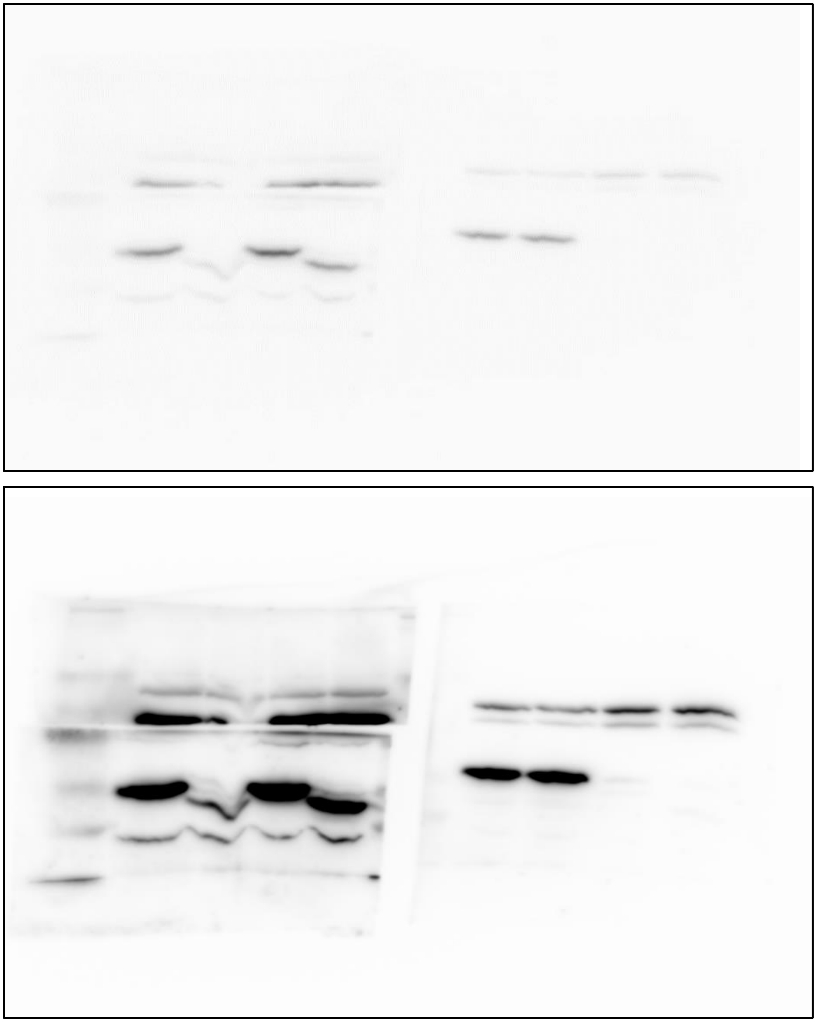

3E THP-1 cells

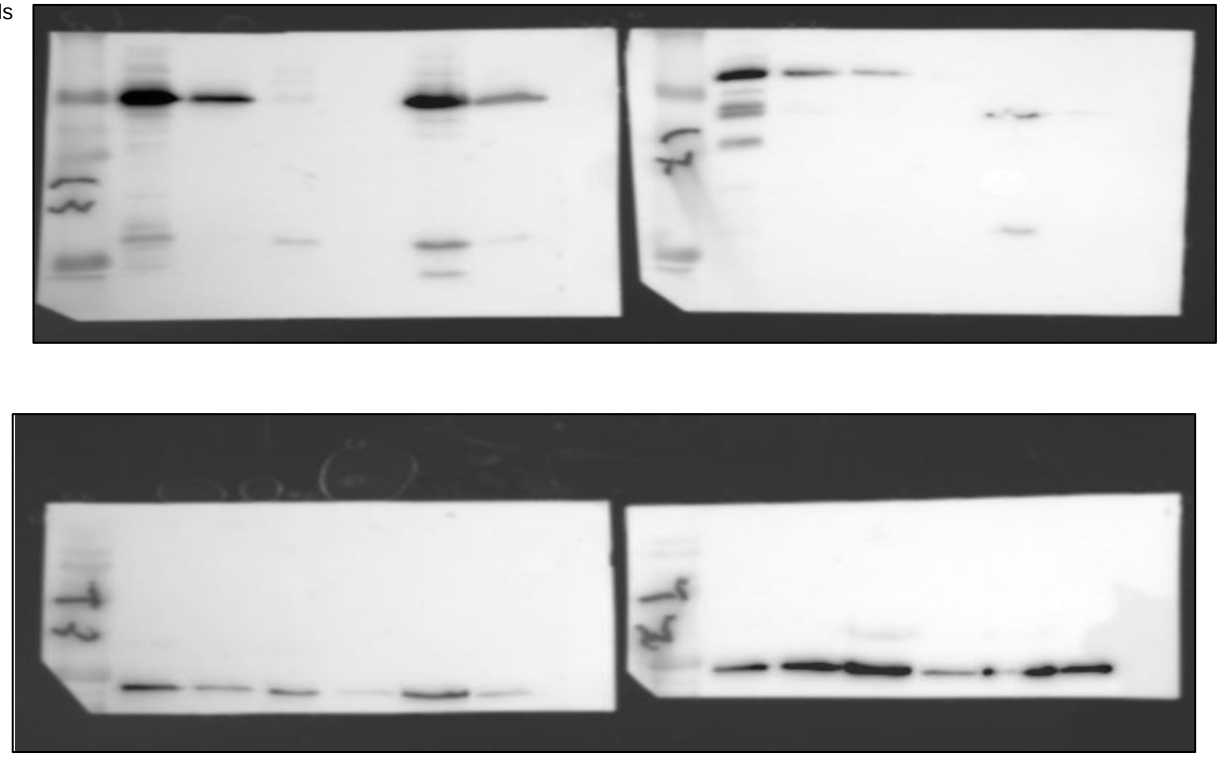

4G

Lm supernatant
αLLO immunoblot

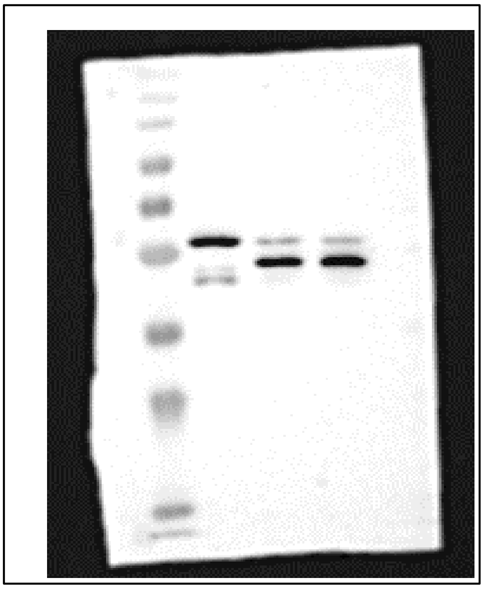

kDa
75
63
48
35
25
15

5A

pIMK2 in ΔLLO:

αLLO

αIAP

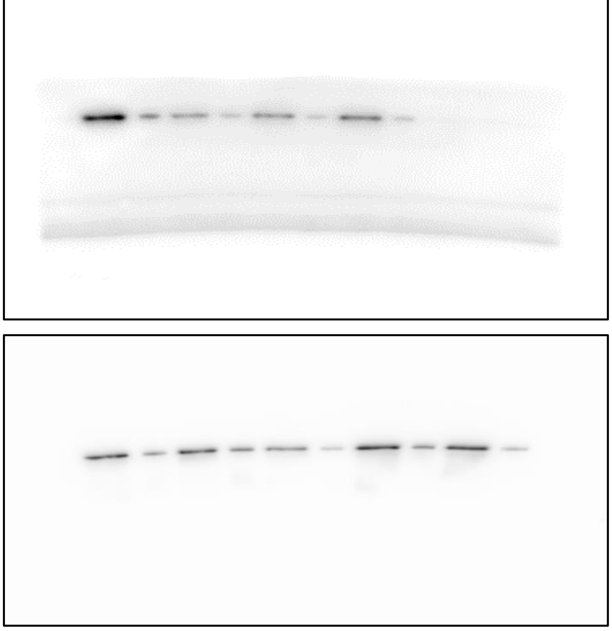

5B

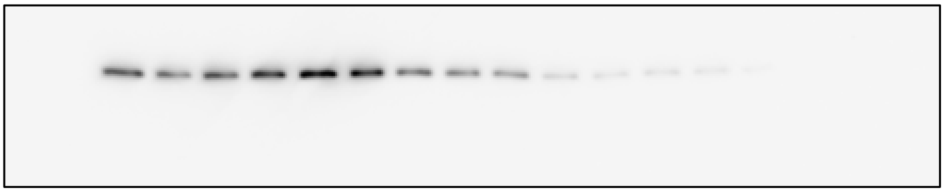

5G

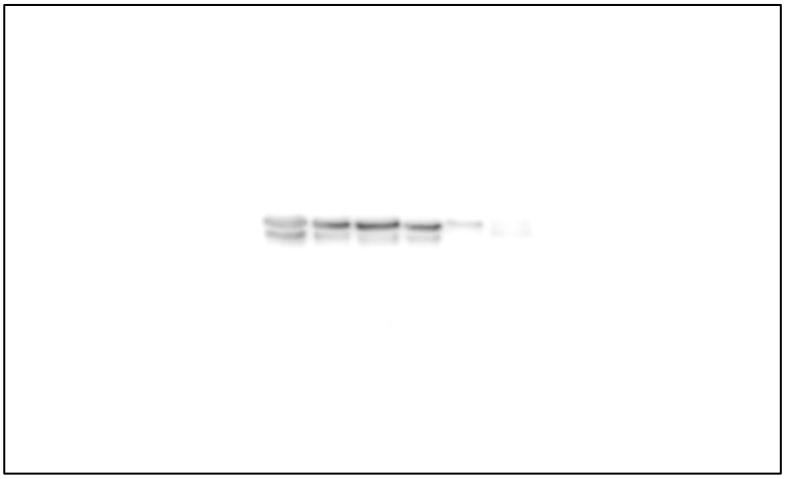

S3B

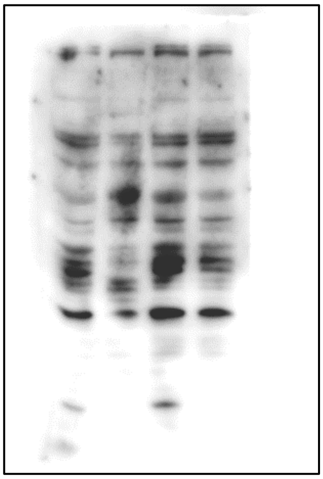

Supplement: Supplementary file 2 — Uncropped western blots [file 41419_2025_7365_MOESM2_ESM.pdf]
